# Single-molecule fluorescence-based approach reveals novel mechanistic insights into human small heat shock protein chaperone function

**DOI:** 10.1074/jbc.RA120.015419

**Published:** 2020-12-10

**Authors:** Caitlin L. Johnston, Nicholas R. Marzano, Bishnu P. Paudel, George Wright, Justin L.P. Benesch, Antoine M. van Oijen, Heath Ecroyd

**Affiliations:** 1 Molecular Horizons and School of Chemistry and Molecular Bioscience, University of Wollongong, Wollongong, New South Wales, Australia; 2 Illawarra Health & Medical Research Institute, Wollongong, New South Wales, Australia; 3 Department of Chemistry, Physical and Theoretical Chemistry, University of Oxford, Oxford, UK

**Keywords:** molecular chaperone, protein aggregation, alphaB-crystallin, chloride intracellular channel 1, total internal reflection fluorescence microscopy, mass photometry, oligomers, protein complexes, αBc, αB-crystallin, CLIC1, chloride intracellular channel 1, FPP, fluorescently-labeled proteins per point, GST, glutathione-S-transferase, I_0_, initial fluorescence intensity, I_s_, fluorescent intensity of each single-photobleaching event, I_s-mean_, mean intensity of a single photobleaching event, OSS, oxygen scavenger system, proteostasis, protein homeostasis, smFRET, single-molecule FRET, SOD1, superoxide dismutase 1, sHsp, small heat shock protein, TIRF, total internal reflection fluorescence

## Abstract

Small heat shock proteins (sHsps) are a family of ubiquitous intracellular molecular chaperones; some sHsp family members are upregulated under stress conditions and play a vital role in protein homeostasis (proteostasis). It is commonly accepted that these chaperones work by trapping misfolded proteins to prevent their aggregation; however, fundamental questions regarding the molecular mechanism by which sHsps interact with misfolded proteins remain unanswered. The dynamic and polydisperse nature of sHsp oligomers has made studying them challenging using traditional biochemical approaches. Therefore, we have utilized a single-molecule fluorescence-based approach to observe the chaperone action of human alphaB-crystallin (αBc, HSPB5). Using this approach we have, for the first time, determined the stoichiometries of complexes formed between αBc and a model client protein, chloride intracellular channel 1. By examining the dispersity and stoichiometries of these complexes over time, and in response to different concentrations of αBc, we have uncovered unique and important insights into a two-step mechanism by which αBc interacts with misfolded client proteins to prevent their aggregation.

Small heat shock proteins (sHsps) are a diverse and ubiquitously expressed family of intracellular molecular chaperones that play a critical role in the maintenance of protein homeostasis (proteostasis). One of the main roles of sHsps is to bind and trap misfolded proteins to protect cells from irreversible protein aggregation during periods of cellular stress (1, 2, 3). Consequently, sHsp malfunction has been implicated in a number of diseases including cataracts, cancer, motor neuropathies, and neurodegeneration (4, 5, 6).

Typically sHsps form oligomeric species in solution, and this is thought to be linked to their chaperone function. For example, human alphaB-crystallin (αBc: HSPB5), an archetypal sHsp and one of the most widely expressed of the 10 human sHsp isoforms, forms large, polydisperse oligomeric ensembles in dynamic equilibrium mediated by subunit exchange (7, 8, 9). These large oligomers are formed from monomeric and/or dimeric building blocks. Many factors, including the presence of client proteins, temperature, and post-translational modifications, shift the equilibrium from larger polydisperse oligomers to predominantly smaller oligomers, which have been reported to have enhanced chaperone activity (10, 11, 12, 13, 14, 15).

It is well established that sHsps can form complexes with misfolded clients to prevent their aggregation (16, 17, 18). Studies of monodisperse sHsps from plants, using techniques that include size exclusion chromatography, electron microscopy, and native mass spectrometry, have provided important stoichiometric and mechanistic information on the end-stage complexes that these sHsps form with client proteins (19, 20, 21, 22, 23, 24). However, very little is known about the complexes formed between polydisperse mammalian sHsp isoforms and their clients. It has been postulated that for polydisperse sHsps, the initial encounter with client proteins is mediated by smaller sHsp oligomers, which have enhanced chaperone activity as a result of increased exposed hydrophobicity and, therefore, a greater affinity for misfolded and aggregation-prone proteins (25, 26, 27, 28). Nevertheless, the initial encounter of an sHsp with an aggregation-prone client protein has never been observed. Thus, it remains unclear precisely how sHsps capture misfolded proteins to form the sHsp–client complexes observed as a result of their chaperone action.

Single-molecule fluorescence techniques overcome some of the difficulties of studying dynamic and heterogeneous systems by facilitating the observation of individual protein–protein interactions. Consequently, such approaches may be advantageous for the study of molecular chaperones (29, 30) since, in the case of sHsps, they may enable the intial steps of binding with client proteins to be observed and therefore the molecular mechanism of chaperone action of sHsps to be revealed. Thus, in this work we have exploited a single-molecule fluoresence-based assay in order to directly observe complexes formed between αBc and a model client protein, the chloride intracellular channel 1 (CLIC1) protein.

We demonstrate that αBc inhibits the heat-induced amorphous aggregation of CLIC1 and that this inhibitory activity results in the formation of a polydisperse range of αBc–CLIC1 complexes. Employing our single-molecule fluorescence-based assay, we have, for the first time, determined the stoichiometries of complexes formed between αBc and a client protein and measured how these complexes change over time. Our results provide evidence for a two-step mechanism of sHsp–client interaction and provide fundamental insight into the molecular mechanisms by which sHsps interact with client proteins to prevent aggregation as part of proteostasis.

## Results

### CLIC1—a new model client protein for assessing sHsp chaperone activity

CLIC proteins can exist in cells in both a soluble globular form as well as an integral membrane protein with ion channel function (31). The soluble globular form of CLIC1 adopts a glutathione-S-transferase (GST)–like canonical fold and is monomeric (31, 32, 33). We chose to explore CLIC1 as a potential model client protein to study sHsp chaperone function because cytosolic plant sHsps have been shown to bind GST proteins *in vivo* following heat stress (34) and expression of the human sHsp, Hsp27 (HSPB1) protects detoxifying enzymes, such as GSTs, against inactivation in cells (35). Destabilization of CLIC1, whether through a change in pH or temperature, results in the formation of a folding intermediate with a high degree of solvent-exposed hydrophobicity (36, 37), causing it to be decidedly aggregation-prone. This is typical of the client proteins of sHsps that form during times of cellular stress, whereby sHsps bind to these destabilized forms to prevent their aggregation (38). Isoforms of CLIC1 amenable to site-specific labeling at cysteine residues have been previously described (39), including an isoform in which four of the six native cysteines are mutated to alanines (C89A, C178A, C191A, C223A; herein designated CLIC1_cysL_). Together, these characteristics led us to develop CLIC1 as a model client protein for the study of αBc chaperone activity at the single-molecule level.

**Figure 1:**
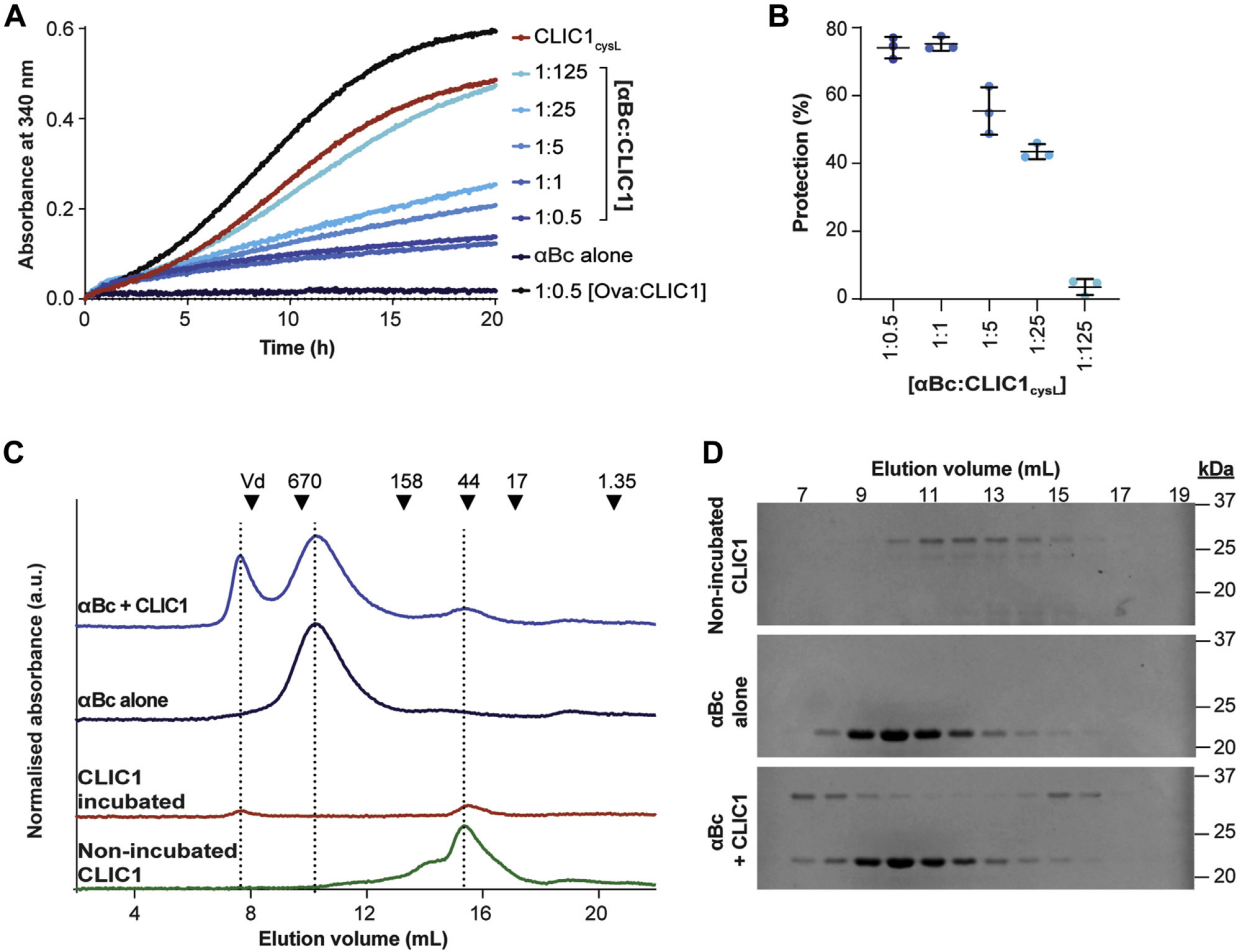
**αBc**_**WT**_**forms high-molecular-mass complexes with CLIC1**_**cysL,**_**inhibiting its amorphous aggregation.***A*, CLIC1_cysL_ (50 μM) was incubated at 37 °C for 20 h in the presence of varying molar ratios of αBc_WT_ (1:125–1:0.5, αBc_WT_–CLIC1) or ovalbumin (Ova). Ovalbumin was used as a non-chaperone control protein at a molar ratio of 1:0.5 (CLIC1–Ova). The aggregation of CLIC1_cysL_ was monitored by measuring the change in light scatter at 340 nm over time. *B*, the percentage protection afforded by varying molar ratios of αBc_WT_ against CLIC1_cysL_ aggregation, reported as the mean ± standard deviation of three independent experiments (n = 3). *C*, size-exclusion chromatograms of non-incubated CLIC1_cysL_ (50 μM) (*green*), and the soluble fraction of samples following incubation; CLIC1_cysL_ in the absence of αBc_WT_ (*red*); αBc_WT_ alone (100 μM, *dark blue*); CLIC1_cysL_ in the of presence αBc_WT_ (*lightblue*, molar ratio 1:0.5, αBc_WT_:CLIC1). *D*, SDS-PAGE of the eluted fractions collected from the size-exclusion column. The elution volume of the fractions is shown at the top of the figure.

We first confirmed that heat destabilization of CLIC1_cysL_ led to its aggregation, akin to the behavior of other client proteins, including luciferase, rhodanese, alcohol dehydrogenase, and malate dehydrogenase, which are typically used to assess chaperone function (40, 41). When CLIC1_cysL_ was incubated at 37 °C, there was a significant increase in light scattering at 340 nm over 20 h, indicative of its destabilization and subsequent aggregation (Fig. 1*A*). However, when CLIC1_cysL_ was incubated in the presence of αBc_WT_, there was a concentration-dependent reduction in the rate and overall amount of light scatter associated with CLIC1_cysL_ aggregation (Fig. 1, *A*–*B*). The specificity of this effect was demonstrated by a negative control (using the non-chaperone protein ovalbumin) not inhibiting the increase in light scatter associated with the aggregation of CLIC1_cysL_. Furthermore, there was no increase in light scatter when αBc_WT_ was incubated alone, demonstrating that the increase in light scatter was exclusively due to the aggregation of CLIC1_cysL_. Analysis by size exclusion chromatography and SDS-PAGE of samples following incubation showed that, when incubated together at a molar ratio of 1:0.5 (αBc_WT_:CLIC1_cysL_), αBc_WT_ and CLIC1_cysL_ coeluted in early fractions (fractions 7–9) from the column, suggesting that αBc_WT_ prevented the heat-induced aggregation of CLIC1_cysL_ *via* the formation of high-molecular mass complexes (Fig. 1, *C*–*D*, Fig. S1). Thus, mild heating at 37 °C leads to the destabilization and aggregation of CLIC1, and αBc_WT_ can inhibit this process by forming complexes with the aggregation-prone protein, demonstrating the utility of CLIC1 as a good model client protein for monitoring molecular chaperone activity.

### Examining the interaction of αBc with CLIC1 *via* single-molecule FRET

**Figure 2:**
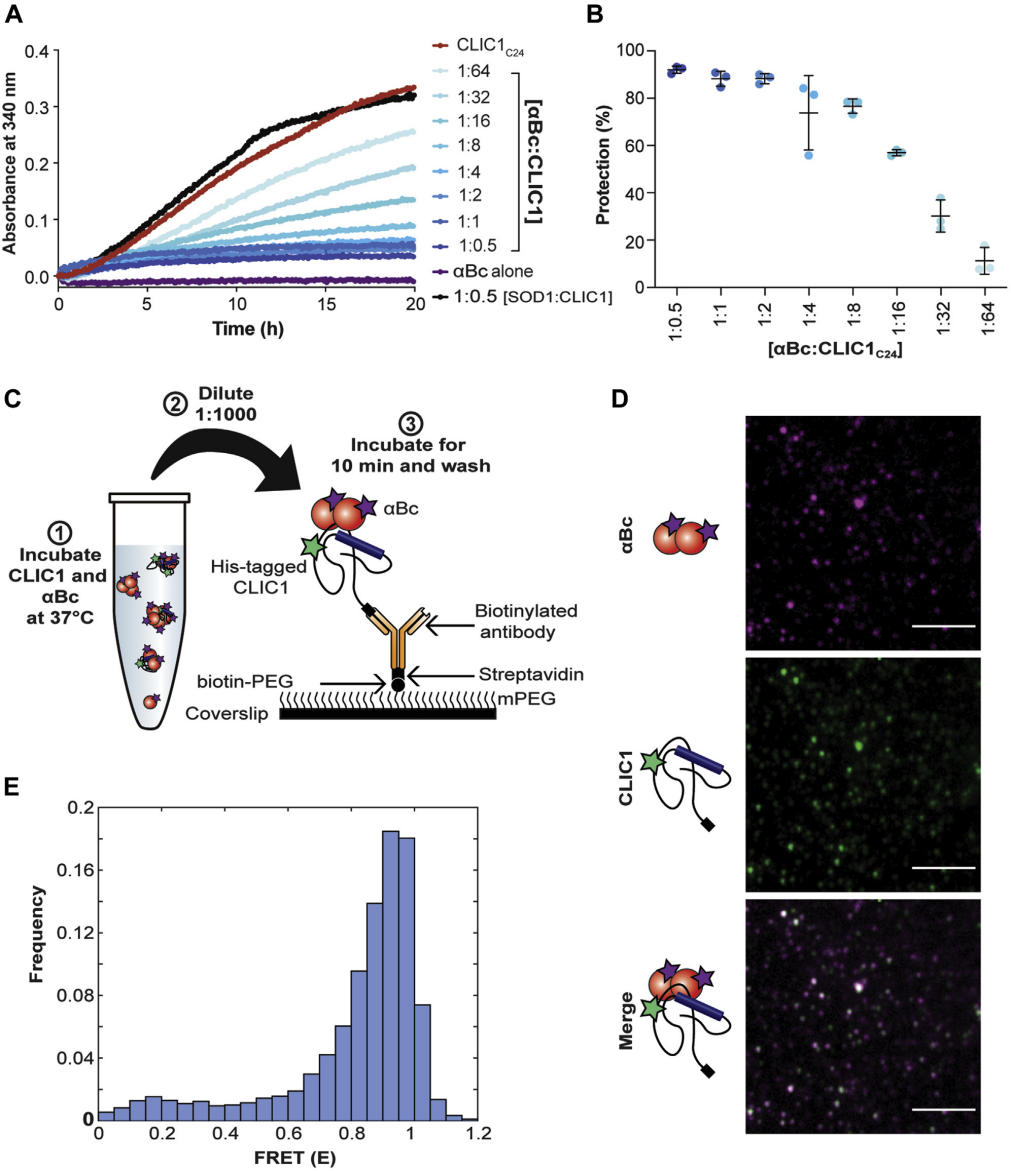
**αBc binds and inhibits the amorphous aggregation of CLIC1**_**C24**_**by forming stable client–chaperone complexes.***A*, a representative aggregation assay performed to assess the ability of αBc_WT_ to inhibit the heat-induced aggregation of CLIC1_C24_. Recombinant CLIC1_C24_ was incubated at 37 °C for 20 h in the presence or absence of varying molar ratios of αBc_WT_ (1:0.5–1:64, αBc_WT_–CLIC1_C24_) or the control protein SOD1. The aggregation of CLIC1_C24_ was monitored by measuring the change in light scatter at 340 nm over time. *B*, the percent inhibition afforded by varying molar ratios of αBc_WT_ against CLIC1_C24_ aggregation, reported as mean ± standard deviation of three independent aggregation assays (n = 3). *C*, schematic of methodology used to form and surface immobilize complexes formed between AF555-CLIC1_C24_ and AF647-αBc_C176_ for smFRET experiments. *D*, representative TIRF microscopy images of AF555-CLIC1_C24_ and AF647-αBc_C176_ complexes. *Scale bar* = 5 μm. *E*, FRET efficiency *(E)* histogram derived from TIRF microscopy data of the initial intensities of CLIC1_C24_–αBc_C176_ complexes prior to photobleaching (n = 421 molecules). αBc, alphaB-crystallin; SOD1, superoxide dismutase 1; TIRF, total internal reflection fluorescence.

To further characterize the nature of the physical interaction between CLIC1 and αBc, we utilized a single-molecule FRET (smFRET)–based approach that allows interactions between biomolecules to be observed (at separations of 2–10 nm). For these experiments, we generated CLIC1_C24_, a CLIC1 isoform that contains a mutation of one of the native tryptophan residues to phenylalanine (W23F) and mutations of five of the native cysteines to alanines (C59A, C89A, C178A, C191A, and C223A); the remaining cysteine (C24) was not modified so it could be exploited for site-specific fluorescent labeling. As observed for CLIC1_cysL_, incubation of CLIC1_C24_ at 37 °C resulted in a significant increase in light scattering at 340 nm over 20 h, indicative of its aggregation, and this was inhibited in a concentration-dependent manner by αBc_WT_, but not the non-chaperone control proteins superoxide dismutase 1 (SOD1: Fig. 2, *A*–*B*) or ovalbumin (Fig. S2*C*). Interestingly, cross-linking of αBc_WT_ had no significant impact on its capacity to inhibit the aggregation of CLIC1_C24_ (Fig. S2, *D*–*F*), suggesting that dynamic subunit exchange of αBc oligomers is not required for the chaperone action in this assay.

To perform smFRET on complexes formed between CLIC1 and αBc, we site-specifically labeled CLIC1_C24_ with an Alexa Flour 555 donor fluorophore. A mutant of αBc (αBc_C176_) was used in these experiments that contains an additional cysteine at the extreme C-terminus of the protein for site-specific attachment of an Alexa Fluor 647 acceptor fluorophore. The addition of the C-terminal cysteine did not affect the ability of the chaperone to inhibit CLIC1_C24_ aggregation (Fig. S2*A*) or substantially change the oligomeric distribution of the protein according to mass photometry measurements (Fig. S3). Mass photometry measurements revealed that while the addition of the fluorescent dye to the C-terminal cysteine did cause a shift in the oligomeric distribution of αBc_C176_ toward smaller species, the protein was still capable of forming larger oligomers. To determine if the fluorescently labeled αBc_C176_ could interact and form client–chaperone complexes with CLIC1_C24_, donor (AF555)-labeled CLIC1_C24_ and acceptor (AF647)-labeled αBc_C176_ were incubated together at 37 °C for 20 h and immobilized on a functionalised coverslip for total internal reflection fluorescence (TIRF) microscopy (Fig. 2*C*). Complexes containing colocalized CLIC1_C24_ and αBc_C176_ were observed at the single-molecule level (Fig. 2*D*), and the approximate time–FRET traces were calculated using the donor and acceptor fluorescence time–intensity traces (Fig. S4*A*). The time-FRET trajectories initially displayed high FRET efficiencies, which gradually decreased over time, likely due to the photobleaching of multiple acceptor fluorophores within the αBc_C176_–CLIC1_C24_ complexes (Fig. S4*B*). Analysis of the initial FRET efficiency of αBc_C176_–CLIC1_C24_ complexes prior to photobleaching showed these complexes had a high FRET efficiency (E = 0.8–1) and therefore were in close proximity, consistent with a stable interaction between αBc_C176_ and heat-destabilized CLIC1_C24_ (Fig. 2*E*). However, the complexity of these smFRET traces, as a result of multiple donor and acceptor fluorophores within the complexes, means calculation of accurate distances between acceptor and donor fluorophores and the precise stoichiometries of αBc_C176_ and CLIC1_C24_ cannot readily be determined using this approach.

### A single-molecule fluorescence-based approach can be used to examine interactions between αBc and CLIC1

**Figure 3:**
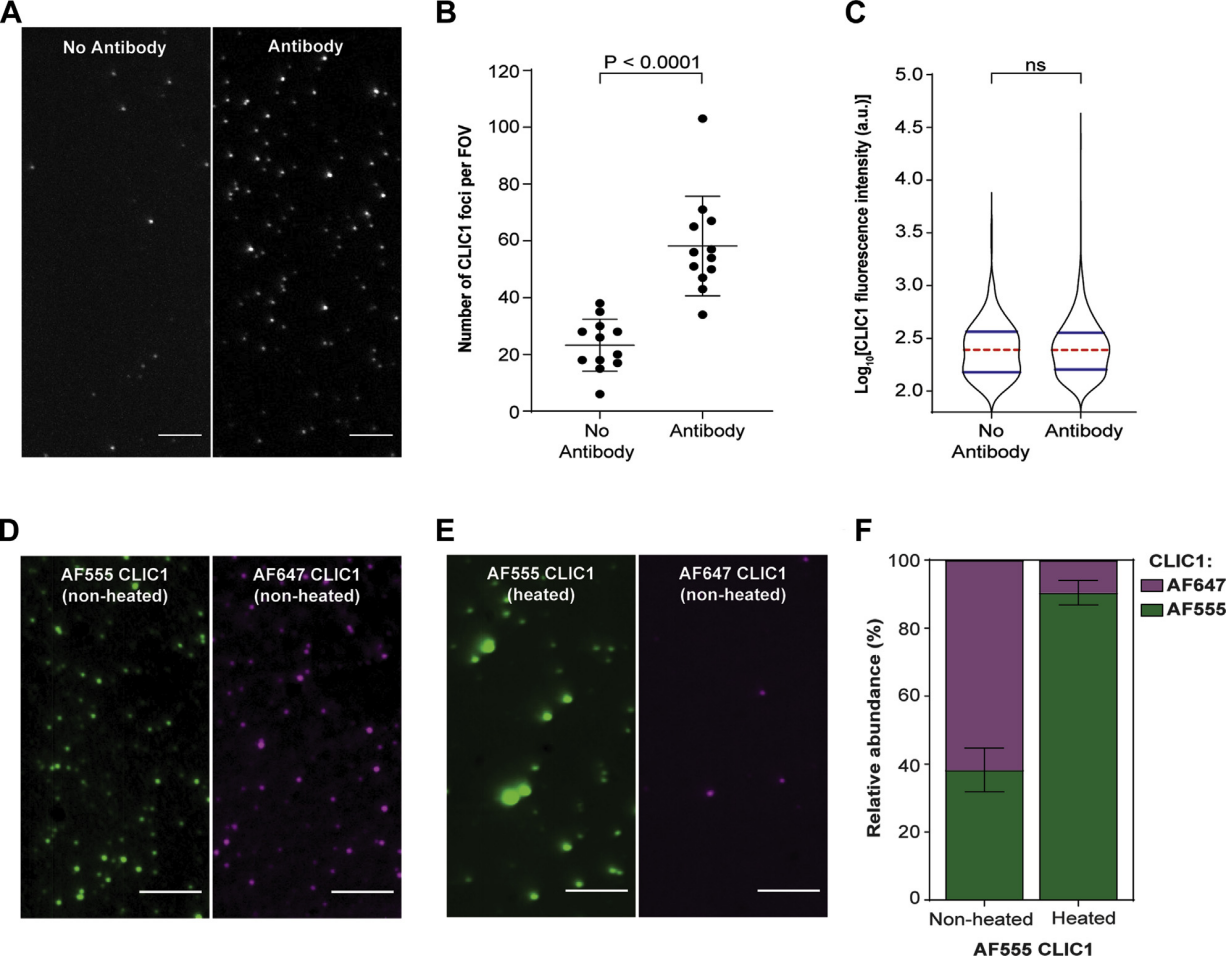
**The binding of CLIC1 to functionalized coverslips for analysis by a single-molecule fluorescence-based approach.***A*–*C*, AF647-labeled CLIC1_C24_ (1 μM) was incubated at 37 °C for 2 h before being diluted 1:1000 into imaging buffer and loaded into flow cells in the presence and absence of a surface-bound anti-6X His-tag antibody. Following a 10-min incubation, flow cells were washed and imaged using TIRF microscopy. *A*, representative images of surface-bound AF647-CLIC_C24_ in the absence (*left*) or presence (*right*) of surface-immobilized antibodies. *Scale bar* = 5 μm. *B*, the number of CLIC1_C24_ foci per field of view (FOV) on coverslips in the presence or absence of the anti-6X His-tag antibody, reported as mean ± standard deviation (n = 12). Comparisons of the treatment groups were performed *via* a student’s *t* test. *C*, violin plots showing the distribution of the fluorescence intensity of AF647-CLIC1_C24_ foci in the presence or absence of the antibody. The plots show the kernel probability density (*black outline*), median (*red*), and interquartile range (*blue*). Comparisons of distributions were performed using the Kruskal–Wallis test for multiple comparisons with Dunn’s procedure. *D*–*F*, AF647-CLIC1_C24_ was incubated in the presence of heated (previously for 2 h at 37 °C) or nonheated AF555-CLIC1_C24_ (1 μM) for 5 min on ice. Samples were diluted 1:1000 and were loaded into flow cells before being washed and imaged using TIRF microscopy. Representative images of surface-bound (*D*) nonheated AF555-CLIC1_C24_ (*green*) and AF647-CLIC1_C24_ (*magenta*) or (*E*) heated AF555-CLIC1_C24_ (*green*) and nonheated AF647-CLIC1_C24_ (*magenta*). *F*, the relative abundance of each fluorescently labeled CLIC1_C24_ species per FOV reported as mean ± standard deviation (n = 15). TIRF, total internal reflection fluorescence.

We hence sought to employ a single-molecule fluorescence-based assay that would enable the stoichiometries of αBc_C176_ and CLIC1_C24_ within complexes to be interrogated. To do so, we first investigated the binding of heated (37 °C for 2 h) site-specific fluorescently labeled CLIC1_C24_ (AF647-CLIC1_C24_) to the surface of a functionalised coverslip (Fig. 3*A*). As expected, there was a significant increase in the number of CLIC1_C24_ foci observed when the capture antibody was present (Fig. 3*B*). Moreover, there was no difference in the fluorescent intensities of the CLIC1_C24_ species bound to the coverslip in the presence or absence of the antibody (Fig. 3*C*), demonstrating that the CLIC1_C24_ bound by the antibody is representative of the CLIC1_C24_ species present in solution. Heated CLIC1_C24_ was immobilized to the functionalized coverslip much more readily than folded CLIC1_C24_ (Fig. 3, *D*–*F*), presumably due to increased exposure of the N-terminal His-tag as a result of CLIC1_C24_ unfolding. Thus, our single-molecule approach efficiently captures the thermally destabilized CLIC1_C24_ species that are potential clients of sHsps.

**Figure 4:**
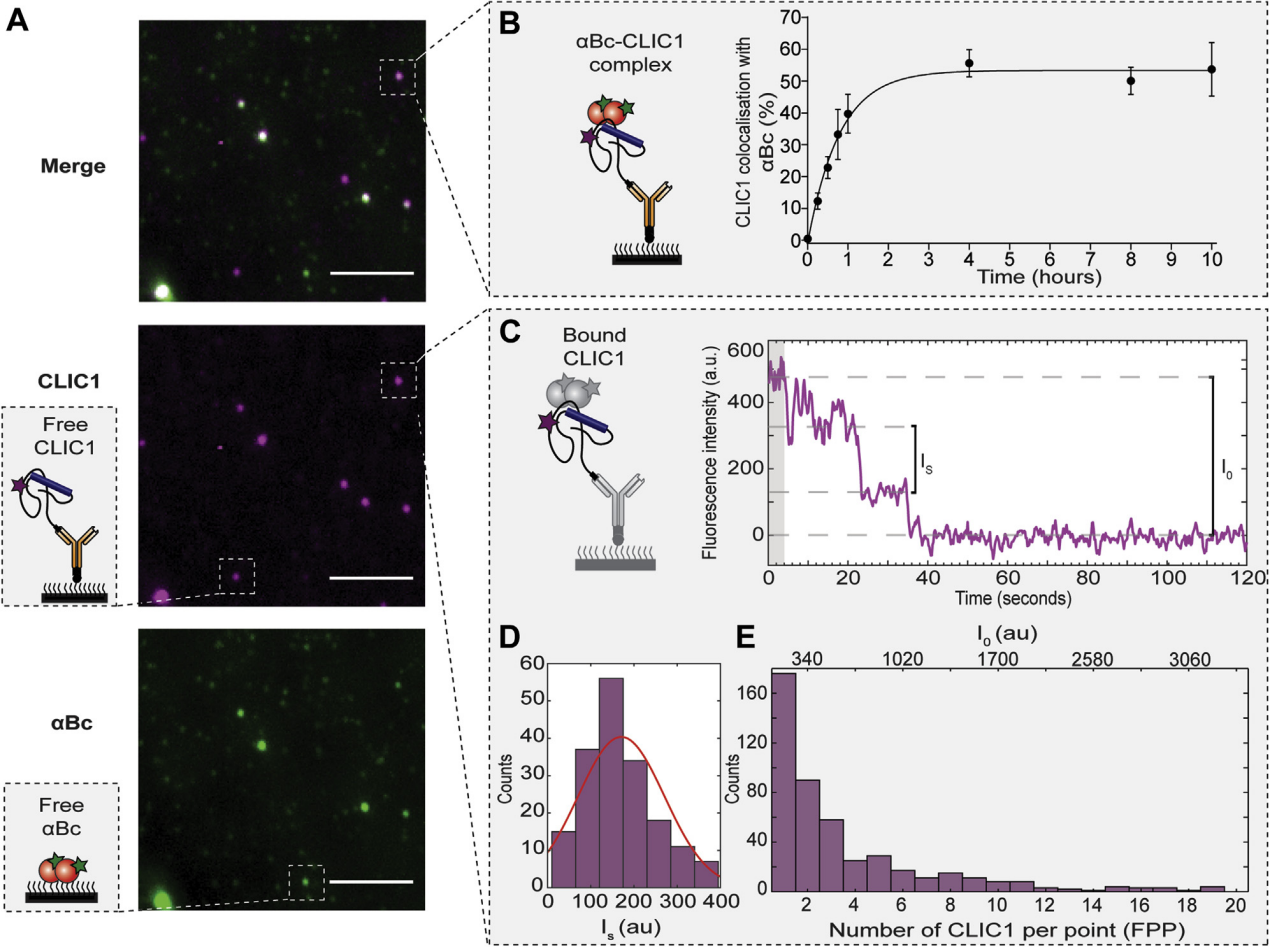
**Characterization of CLIC1**_**C24**_**–αBc**_**C176**_**complexes using a single-molecule fluorescence-based approach.** AF488-αBc_C176_ was incubated with AF647-CLIC1_C24_ (2:1 molar ratio) at 37 °C for 10 h to form complexes. Aliquots were taken at multiple timepoints throughout the incubation for TIRF microscopy imaging. *A*, representative TIRF microscopy images of complexes at 10 h. *Scale bar* = 5 μm. Schematic indicating free CLIC1_C24_ and αBc_C176_ bound to the coverslip surface. *B*, schematic showing the immobilization of αBc_C176_–CLIC1_C24_ complexes to the surface of a glass coverslip. The percentage of CLIC1_C24_ colocalized with αBc_C176_ over time reported as the mean ± standard deviation of three independent experiments. Data were fit using a one-phase association model. *C*, example time trace of the fluorescent intensity of AF647-CLIC1_C24_ in complex with AF488-αBc_C176_. The shaded area (*gray*) represents the first 20 values that were averaged to determine the initial intensity (*I*_*0*_). *D*, photobleaching traces from AF647-CLIC1_C24_ molecules with distinct photobleaching steps were manually identified and fit to a change point analysis to calculate the fluorescent intensity of each single-photobleaching event (*I*_*s*_). The *I*_*s*_ values were fit to a Gaussian distribution to determine the mean intensity of a single photobleaching event (*I*_*s-mean*_). *E*, example histogram of CLIC1_C24_ showing the distribution of *I*_*0*_ and fluorescently labeled proteins per point (FPP) at 10 h. FPP were calculated using the equation *FPP* = *I*_*0*_/*I*_*s-mean*_ for all the CLIC1_C24_ in complex with αBc_C176_. TIRF, total internal reflection fluorescence.

We next incubated AF647-CLIC1_C24_ and AF488-αBc_C176_ together at 37 °C and collected aliquots at various timepoints over a 10-h period. Samples were then diluted and immediately immobilized to the coverslip surface (*via* the His-tag on CLIC1_C24_) for imaging using TIRF microscopy. As expected, αBc_C176_ (green) was observed to colocalize with CLIC1_C24_ molecules (magenta) (Fig. 4*A*), indicative of the formation of stable complexes between these two proteins and consistent with the results of the smFRET experiments (Fig. 2*D*). The proportion of CLIC1_C24_ molecules colocalized with αBc_C176_ increased rapidly over 1 h (Fig. 4*B*). Interestingly, after 4 h, the proportion of CLIC1_C24_ colocalized with αBc_C176_ reached a maximum of approximately 50%, demonstrating that not all CLIC1_C24_ molecules were in complex with αBc_C176_ under these experimental conditions (these CLIC1_C24_ molecules not in complex with αBc_C176_ are herein referred to as free CLIC1_C24_ species). Additionally, despite having blocked (passivated) the coverslip surface, which significantly reduced the nonspecific binding of αBc_C176_ to the coverslip, some nonspecific binding of αBc_C176_ molecules not in complex with CLIC1_C24_ was also observed (herein referred to as free αBc_C176_ species) (Fig. 4*A*). Negative stain transmission electron microscopy (TEM) demonstrated the heterogeneous nature of the AF488-αBc_C176_ oligomers and the species present following incubation of AF647-CLIC1_C24_ and AF488-αBc_C176_ at 37 °C (Fig. S5). This heterogeneity precluded any detailed analysis of complexes formed between CLIC1_C24_ and αBc_C176_ *via* TEM; however, we did observe an apparent reduction in the size of species in samples containing both CLIC1_C24_ and αBc_C176_ compared with those containing only αBc_C176_.

To determine the stoichiometries of CLIC1_C24_ and αBc_C176_ in complexes formed under conditions in which CLIC1_C24_ is prone to aggregation, molecules were imaged until all fluorophores were completely photobleached. CLIC1_C24_ and αBc_C176_ trajectories with distinct photobleaching steps were identified manually and used to calculate the fluorescent intensity of each single-photobleaching event (*I*_*s*_) (Fig. 4*C*, Figs. S6, *A* and *D* and S7*B*). The *I*_*s*_ values collected from CLIC1_C24_ and αBc_C176_ trajectories containing one distinct photobleaching step were not significantly different when the proteins were in a complex or alone (αBc_C176_ nonspecifically bound to the surface was used to assess the protein when not in a complex) (Fig. S6, *B* and *E*). Therefore, binding of the two proteins into a complex did not significantly affect the fluorescent intensity of the fluorophores attached to the proteins. Analysis of trajectories from CLIC1_C24_–αBc_C176_ complexes that contained multiple distinct photobleaching steps showed a broader distribution of *I*_*S*_ values than complexes containing only a single unit of either protein (Fig. S6, *B* and *E*). Thus, to establish the number of CLIC1_C24_ or αBc_C176_ in complexes, *I*_*s*_ values calculated from trajectories with multiple distinct photobleaching steps were fit to a Gaussian distribution from which the mean intensity of a single photobleaching event (*I*_*s-mean*_) for CLIC1_C24_ or αBc_C176_ was derived (Fig. 4*D*, Fig. S7*C*). The *I*_*s-mean*_ values determined using change point analysis were 170.5 ± 99 a.u and 166 ± 119 a.u for CLIC1_C24_ and αBc_C176_, respectively. These values were then used to determine the number of fluorescently labeled proteins per point (*FPP*). The initial fluorescence intensities (*I*_*0*_) for CLIC1_C24_ and αBc_C176_ in each complex were calculated by averaging the first 20 intensity values (Fig. 4*C*, Fig. S7*B*). Change point analysis was not used to calculate *I*_*0*_ owing to its inability to accurately fit photobleaching steps of larger complexes (*i.e.*, >10mers). Furthermore, calculation of *I*_*0*_ *via* either change point analysis or averaging of the initial 20 intensity values of trajectories yielded similar values when used to analyze CLIC1_C24_ and αBc_C176_ trajectories (<10mers) with multiple distinct photobleaching steps (Fig. S6, *C* and *F*). Subsequently *I*_*0*_ for CLIC1_C24_ and αBc_C176_ in each complex was divided by the appropriate *I*_*s-mean*_ to calculate the *FPP*. These *FPP* values were then used to determine the number of subunits of each protein present in complexes of up to a maximum of 20 subunits (Fig. 4*E*, Fig. S7*D*; see Two-color TIRF microscopy data and statistical analysis in the Experimental procedures section).

To investigate whether the dilution and immobilization of αBc_C176_–CLIC1_C24_ complexes required for this single-molecule fluorescence approach affected the nature of the complexes formed at higher concentrations, complexes were cross-linked prior to dilution and single-molecule measurements (Fig. S8). When αBc_C176_ was cross-linked in the absence of CLIC1_C24_ and diluted for TIRF microscopy, a small decrease in the αBc_C176_ oligomer size indicative of some dissociation of large oligomers was observed (Fig. S8, *A*–*C*). However, this decrease in the size of αBc_C176_ oligomers was not observed when it was in complex with CLIC1_C24_ (Fig. S8, *E*, *G* and *H*). Furthermore, the size and amount of CLIC1_C24_ in complex with αBc_C176_ was not significantly affected by dilution and immobilization of the complexes (Fig. S8, *E*, *G* and *H*), indicating that the complexes observed by single-molecule fluorescence imaging are representative of those formed during the incubation at 37 °C. However, when comparing the size distributions of αBc_C176_ observed by this single-molecule fluorescence approach with those obtained by mass photometry, it is apparent that the single-molecule fluorescence approach primarily detects the smaller oligomers (<10 subunits) (Fig. S8*D*).

### The size and polydispersity of complexes formed between αBc and CLIC1 increase over time

**Figure 5:**
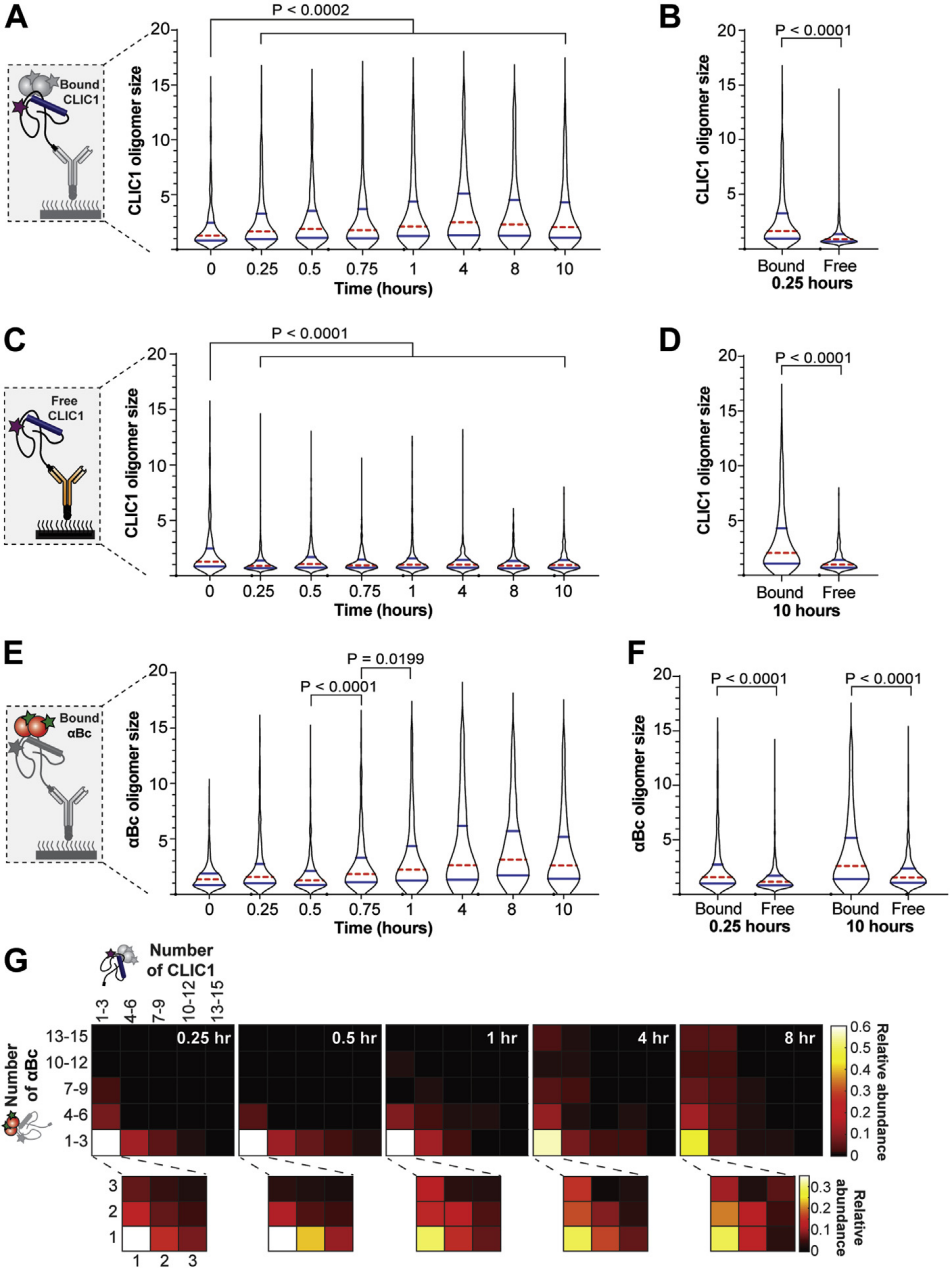
**αBc**_**C176**_**-CLIC1**_**C24**_**complexes increase in polydispersity and size over time.** AF488-αBc_C176_ was incubated with AF647–CLIC1_C24_ (2:1 molar ratio) at 37 °C for 10 h, with aliquots taken at multiple timepoints throughout the incubation. Following incubation, aliquots were immediately diluted and incubated in flow cells for 10 min before being washed and imaged using TIRF microscopy. Violin plots showing the size distribution over 10 h at 37° of (*A*) free CLIC1_C24_ that is not in complex with αBc_C176_, (*B*) CLIC1_C24_ bound to αBc_C176_ or free CLIC1_C24_ after 0.25 h of incubation, (*C*) CLIC1_C24_ bound to αBc_C176_, (*D*) CLIC1_C24_ bound to αBc_C176_ or free CLIC1_C24_ after 10 h of incubation, (*E*) αBc_C176_ bound to CLIC1_C24_, and (*F*) αBc_C176_ bound to CLIC1_C24_ or nonspecifically adsorbed to the surface (Free) after 10 h. The violin plots show the kernel probability density (*black outline*), median (*red*), and interquartile range (*blue*). Results include measurements from three independent experiments (n = 3), and comparisons of distributions were performed using the Kruskal–Wallis test for multiple comparisons with Dunn’s procedure (*p* values indicated). *G*, heatmaps showing the relative abundance of αBc_C176_–CLIC1_C24_ complexes and their stoichiometries over 8 h of incubation. TIRF, total internal reflection fluorescence.

To obtain further information on the interaction between αBc_C176_ and CLIC1_C24_, we examined the change in size and composition of the αBc_C176_–CLIC1_C24_ complexes over time, as well as the size of the molecules that were not in complex. Prior to incubation, both CLIC1_C24_ and αBc_C176_ were present predominantly as smaller noncolocalized species (Fig. 5, *A* and *E*). Following incubation at 37 °C for 0.25 h, αBc_C176_ was found bound to oligomeric species of CLIC1_C24_ that were significantly larger in size than free CLIC1_C24_ species (Fig. 5*B*, *p* < 0.0001). After 0.25 h of incubation, both the bound and free CLIC1_C24_ oligomers did not increase in size (Fig. 5, *A* and *C*). Moreover, the CLIC1_C24_ species not in complex were significantly smaller than the bound species throughout the entire incubation period (Fig 5*D*). Interestingly, following incubation, the size of the noncomplexed CLIC1_C24_ significantly decreased (*p* < 0.001), such that by 10 h primarily monomers were present. This suggests that CLIC1_C24_ species larger than monomers were preferentially bound by αBc_C176_ upon heating.

During the early stages of the incubation (up to 0.5 h), αBc_C176_ in complex with CLIC1_C24_ was primarily monomeric or dimeric (Fig. 5*E*). However, after 0.5 h of incubation, the number of αBc_C176_ molecules in these complexes significantly increased over time, reaching a maximum after 1 h. Analysis of nonspecifically adsorbed αBc_C176_ species indicated that they were significantly smaller in size than αBc_C176_ that was in complex with CLIC1_C24_ throughout the incubation period (*p* < 0.0001; Fig. 5*F*, Fig. S9).

We next utilized our single-molecule fluorescence-based approach to characterize the stoichiometries of αBc_C176_–CLIC1_C24_ in individual complexes and interrogate how these change as a function of incubation time. For each individually identified αBc_C176_–CLIC1_C24_ complex, we determined the αBc_C176_–CLIC1_C24_ stoichiometry by calculating the number of monomers of each protein present. This process allowed us to quantify the relative abundance of these stoichiometries over time. Interestingly, we observed that complexes became increasingly polydisperse over the observation time (Fig. 5*G*). At early timepoints during the incubation (0.25–0.5 h), complexes were comprised predominantly of smaller species of αBc_C176_ (monomers-3mers) bound to a polydisperse range of CLIC1_C24_ oligomers (monomers to 12mers). The most abundant complex observed was comprised of monomeric αBc_C176_ bound to a single subunit of CLIC1_C24_. The polydispersity of CLIC1_C24_ within complexes (monomers to 12mers) did not change greatly over 8 h; however, the relative abundance of complexes with more αBc_C176_ (>6mers) increased after 1 h. This increase in the number of αBc_C176_ monomers present in complexes was consistent with the observed increase in the size distribution of αBc_C176_ over time (Fig. 5*E*). Together, these results suggest smaller αBc_C176_ subunits initially bind to aggregation-prone CLIC1_C24_ to form chaperone–client complexes and, over time, additional free αBc_C176_ subunits bind to these complexes until the system reaches equilibrium.

### Chaperone concentration influences the stoichiometries of CLIC1–αBc complexes

**Figure 6:**
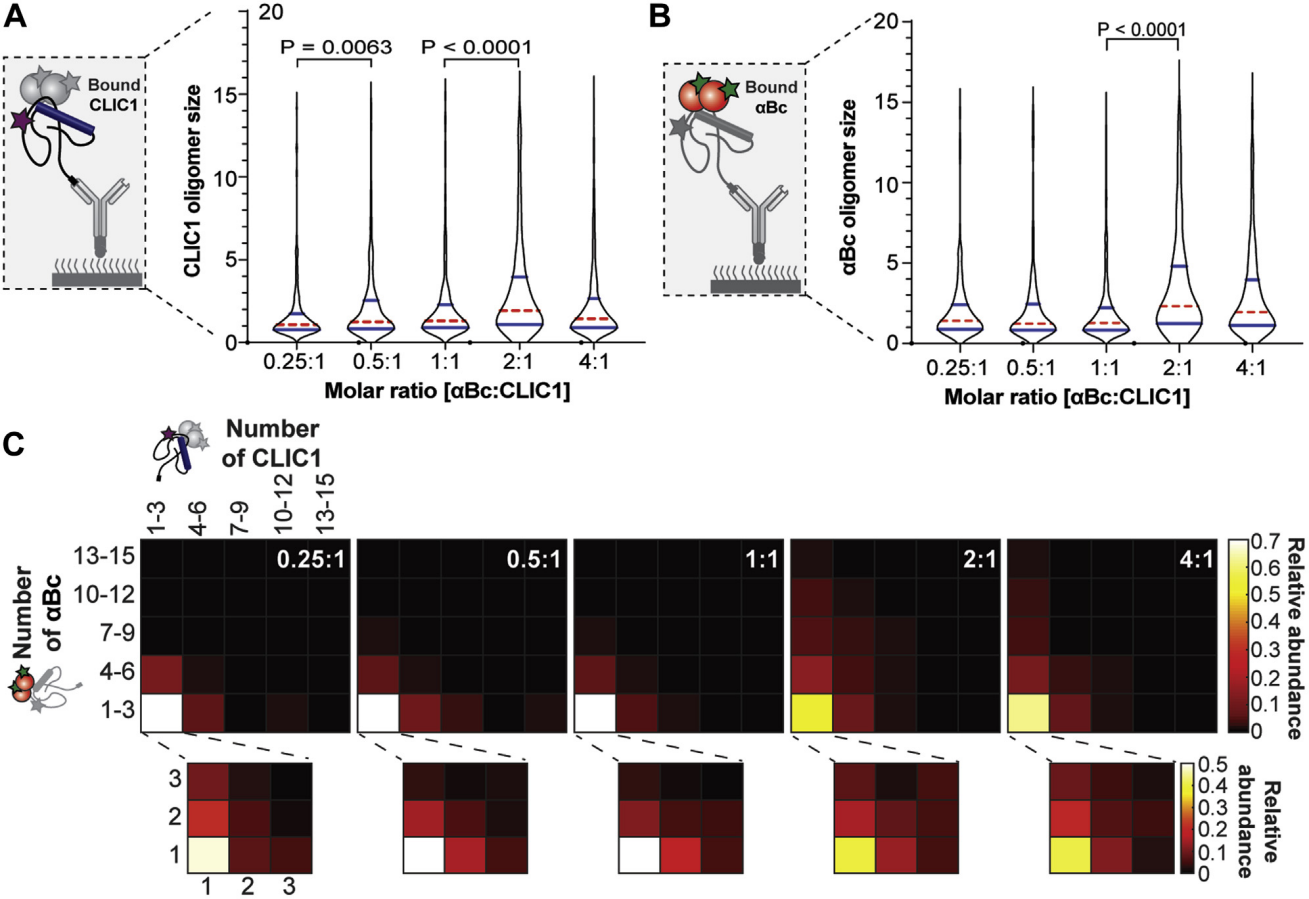
**αBc**_**C176**_**–CLIC1**_**C24**_**complexes change in size and stoichiometry with increasing αBc**_**C176**_**concentration.** AF647-CLIC1_C24_ was incubated in the presence of varying molar ratios of AF488-αBc_C176_ at 37 °C for 8 h. Following incubation, samples were immediately diluted and incubated in flow cells for 10 min before being washed and imaged using TIRF microscopy. The size distributions of CLIC1_C24_ (*A*) in complex with αBc_C176_ (*B*) at increasing molar ratios of αBc_C176_–CLIC1_C24_. The violin plots show the kernel probability density (*black outline*), median (*red*), and interquartile range (*blue*). Result are representative of two independent experiments (n = 2), and comparisons of distributions were performed using the Kruskal–Wallis test for multiple comparisons with Dunn’s procedure (*p* values indicated). *C*, heatmaps showing the relative abundance of αBc_c176_–CLIC1_C24_ complexes with increasing molar ratios of αBc_C176_–CLIC1_C24_. TIRF, total internal reflection fluorescence.

The molar ratio of sHsp to client protein is thought to be one of the most important parameters that determines the nature and size of sHsp–client complexes (18, 19, 20, 21, 23, 42, 43). Therefore, we exploited our single-molecule fluorescence assay to investigate how sHsp concentration affects the stoichiometries of complexes formed with CLIC1_C24_. We observed that the size of CLIC1_C24_ species in complex with αBc_C176_ significantly increased with increasing relative amounts of αBc_C176_ (molar ratios from 0.25:1 to 4:1, αBc_C176_–CLIC1_C24_) (Fig. 6*A*). Conversely, the number of αBc_C176_ subunits in complexes was significantly smaller (*p* < 0.0001) when the sHsp was present at a molar ratio below or equal to the amount of CLIC1_C24_ present (0.25:1–1:1, αBc_C176_–CLIC1_C24_) (Fig. 6*B*). The number of αBc_C176_ subunits in complexes significantly increased when the αBc_C176_ was present in excess of CLIC1_C24_ (2:1 and 4:1, αBc_C176_–CLIC1_C24_) (Fig. 6*B*). At all molar ratios tested, both αBc_C176_ and CLIC1_C24_ were significantly larger when in complex than when they were not in complex (Fig. S10, *B*–*E*). Interestingly, noncolocalized αBc_C176_ was observed to be significantly larger in size when incubated at the higher concentrations (>1 μM) used in these experiments (Fig. S10*D*).

As observed previously, the complexes formed between αBc_C176_ and CLIC1_C24_ after heating were heterogeneous (Fig. 6*C*). Examination of the relative abundance of complexes formed when the molar ratio of αBc_C176_–CLIC1_C24_ was low ([0.25:1]–[1:1]) indicated that a small number of αBc_C176_ subunits (monomers-6mers) were in complex with CLIC1_C24_ species (monomers-6mers). In contrast, when complexes were formed at higher molar ratios of αBc_C176_–CLIC1_C24_ ([2:1]–[4:1]), although the number of CLIC1_C24_ species within the complexes did not change (monomers-6mers), the complexes did increase in the number of αBc_C176_ subunits (>10mers). Consequently, these data suggest that higher concentrations of αBc_C176_ result in an increased binding of free αBc_C176_ subunits to the initial complexes that are formed with CLIC1_C24_.

## Discussion

In this study, we set out to detect and quantify for the first time the initial binding events between an sHsp and client protein. To do so, we employed single-molecule fluorescence assays to study the chaperone action of αBc, an archetypal mammalian sHsp. By employing this single-molecule fluorescence-based approach, we have determined the stoichiometries of complexes formed between αBc and a client protein, CLIC1. From examination of the polydispersity and stoichiometries of these complexes over time, we have uncovered unique and important insights into the mechanism by which αBc captures misfolded client proteins to prevent their aggregation.

The most commonly used approach to investigate chaperone activity is assays that monitor the aggregation of proteins *in vitro*, *via* either light scatter or, in the case of amyloid fibril formation, fluorescent dyes such as Thioflavin T (44). We exploited CLIC1 as a model client protein in this work since it has been previously shown that sHsps interact with proteins with a GST fold in heat-stressed plants (34) and destabilization of CLIC1 results in it forming a folding intermediate with a high degree of solvent-exposed hydrophobicity (36, 37), which is typical of sHsp client proteins that form during cellular stress. Indeed, we demonstrate *via* a light scattering assay that mild heating at 37 °C leads to the aggregation of the CLIC1 isoforms used in this work. Moreover, αBc is able to effectively inhibit this heat-induced aggregation of CLIC1 by forming complexes with it. However, these bulk ensemble assays struggle to provide mechanistic details concerning the interactions that occur between the chaperone and client protein which result in the suppression of aggregation. Approaches such as size exclusion chromatography, electron microscopy, and native mass spectrometry have traditionally been used to examine the end-stage complexes formed between sHsps and client proteins. However, these approaches are limited in their ability to capture the initial binding events between sHsps and client proteins and the dynamic and heterogeneous nature of these complexes. In order to overcome these limitations, we employed a single-molecule fluorescence-based approach that, by utilizing a step-wise photobleaching method, enables the stoichiometries of the chaperone–client complexes in solution to be revealed. In the case of αBc and CLIC1, by monitoring complexes in solution through time, we have been able to uncover novel details of how this sHsp forms complexes with client proteins.

By using mass photometry, we demonstrated that unlabeled αBc was present as two distinct populations of large (20–40mers) and smaller (<10mers) species at the concentrations used to form complexes with CLIC1 for the analysis by single-molecule fluorescence, consistent with previous studies examining the oligomeric distribution of αBc (8). The mass photometry measurements revealed that addition of the C-terminal cysteine caused little change to the oligomeric distribution of αBc other than a slightly higher proportion of oligomers in the range of 400 to 600 kDa. Incorporation of the fluorescent dye onto this cysteine residue resulted in an increase in the proportion of small αBc oligomers; however, large oligomers still formed in this sample and the protein was still chaperone active. Comparison of the size distribution of αBc obtained *via* mass photometry and the single-molecule fluorescence-based approach indicates that the latter is uniquely able to primarily detect small oligomeric species formed by αBc. This is presumably because these smaller species more readily interact with the coverslip surface used in the single-molecule fluorescence assay, possibly because they have increased amounts of exposed charged and polar residues (15). Moreover, cross-linking of αBc demonstrated that there is also some dissociation of large oligomers as a result of the 1000-fold dilution required for single-molecule fluorescence analysis. In addition, since our single-molecule fluorescence technique is unable to accurately determine the size of αBc oligomers that contain more than 20 subunits, it is limited in its ability to characterize some of the very large oligomers and complexes formed by this sHsp. However, given that the smaller oligomeric species of sHsps have been reported to have enhanced chaperone activity (11, 12, 13, 14), proposed to be as a result of increased surface hydrophobicity and dynamism in these dissociated forms (15, 45), our single-molecule fluorescence-based approach is well suited to examining the initial binding events between these small sHsp oligomers and aggregation-prone proteins.

Importantly, the single-molecule methods (mass photometry and single-molecule fluorescence) we have used to describe the oligomeric distribution of αBc involve counting single particles, *i.e.*, a 40-mer oligomer gives the same count (1) as a dimer (1), even though the 40-mer contains 20-times more monomeric subunits. This contrasts to techniques typically used to assess the oligomeric distribution of αBc, such as size-exclusion chromatography (SEC) or analytical ultracentrifugation, which rely on measuring UV absorbance to detect species; thus, using these techniques a single 40-mer gives an absorbance 20-fold higher than a single dimer. This needs to be considered when comparing the relative abundances of protein complexes obtained using these single-molecule techniques with those obtained *via* techniques such as SEC and analytical ultracentrifugation.

Our single-molecule fluorescence data show that the end-stage complexes formed between αBc and CLIC1 are highly heterogeneous, a finding confirmed by TEM analysis of these samples. By examining how these end-stage complexes form, we demonstrate that initially smaller species of αBc (predominantly monomers and dimers) bind to heat-destabilized CLIC1 oligomers. Using this single-molecule approach, we are unable to specifically determine whether there are differences in the binding capacity of small and large oligomers. Nonetheless, our observations validate previous suggestions, based on studying end-stage complexes, that smaller species of sHsps have high chaperone ability and can bind to misfolded proteins (15, 28, 46). Interestingly, we observed that the number of complexes formed between αBc and CLIC1 increased rapidly over the first hour of incubation and reached a plateau after 4 h. During this period, there was an increase in the number of αBc subunits in each αBc–CLIC1 complex. We rationalize this as the recruitment of free αBc subunits onto existing αBc–CLIC1 complexes over time, as has been suggested to occur for other sHsp–client protein interactions (23, 43, 47). Interestingly, we found that prior cross-linking of αBc_C176_ did not significantly impact its capacity to inhibit the heat-induced aggregation of CLIC1_C24_, suggesting that dynamic subunit exchange of αBc oligomers is not required for this chaperone activity and that the additional αBc subunits recruited to existing αBc–CLIC1 complexes do not need to arise as a result of dissociation from larger oligomers.

Varying the molar ratio between CLIC1 and αBc, such that more αBc subunits were available to bind to CLIC1, resulted in an increase in the size of these complexes. We observed a time- and concentration-dependent recruitment of free αBc subunits onto existing αBc–CLIC1 complexes. The lower concentrations of αBc used to form complexes for the single-molecule analyses (2 μM) account for the smaller size of the αBc–CLIC1 complexes detected using this technique compared to the high-molecular-mass complexes observed *via* SEC analysis of samples following the light scattering assay (in which αBc was present at 100 μM). Once formed, cross-linking of the αBc–CLIC1 complexes demonstrates that, upon dilution down to the nM range required for the single-molecule analysis, αBc more readily dissociates from larger sHsp oligomers than from complexes it forms with CLIC1. This is evidenced by our data showing no difference in the size of cross-linked and non-cross-linked αBc–CLIC1 complexes but a decrease in the size of cross-linked and non-cross-linked αBc oligomers. This suggests that the affinity of αBc to destabilized CLIC1 is higher than the affinity of αBc to other αBc subunits. Moreover, this indicates that the observed accumulation of αBc onto αBc–CLIC1 complexes is regulated by the association and dissociation rates of αBc subunits into these complexes and that the dissociation rates from complexes are slower than the timescale of our observations. Hence, αBc subunits are stabilized by the presence of the client protein, a finding supported by the TEM data showing that overall the species formed when αBc is incubated with heat-destabilized CLIC1 are smaller than αBc oligomers. In both prokaryotic (IbpA and IbpB) (47) and eukaryotic sHsp systems (Hsp18.1 and Hsp16.6) (20), sHsp–client complexes are dynamic in that sHsp subunits associate and dissociate from these complexes. Whilst we did not specifically probe for these dynamics in this study, the ability of single-molecule fluorescence techniques to observe dynamic and transient interactions in real time provides the potential to further develop the approaches we have described here in order to examine if dynamic sHsp subunit exchange occurs on sHsp–client protein complexes.

The binding of monomeric αBc to monomers of CLIC1 did not greatly affect the *I*_*s*_ values, suggesting that the photophysical properties of the dye, such as quantum yield, are largely unaffected by the formation of complexes. However, we did observe that oligomers of αBc and CLIC1 in complex displayed a broader distribution of *I*_*s*_ values, suggesting modest effects of the increased heterogeneity and size of the complex on dye intensity. Therefore, whilst we do observe a small proportion of larger αBc–CLIC1 complexes following incubation, these complexes may be under-represented in our data owing to the variability in the emission intensity of the fluorophores attached to αBc or CLIC1 within these larger complexes. Furthermore, these complexes may also be under-represented in our data owing to the His-tag of the CLIC1, which is required for immobilization, possibly becoming buried during the aggregation and/or binding of multiple αBc subunits.

**Figure 7:**
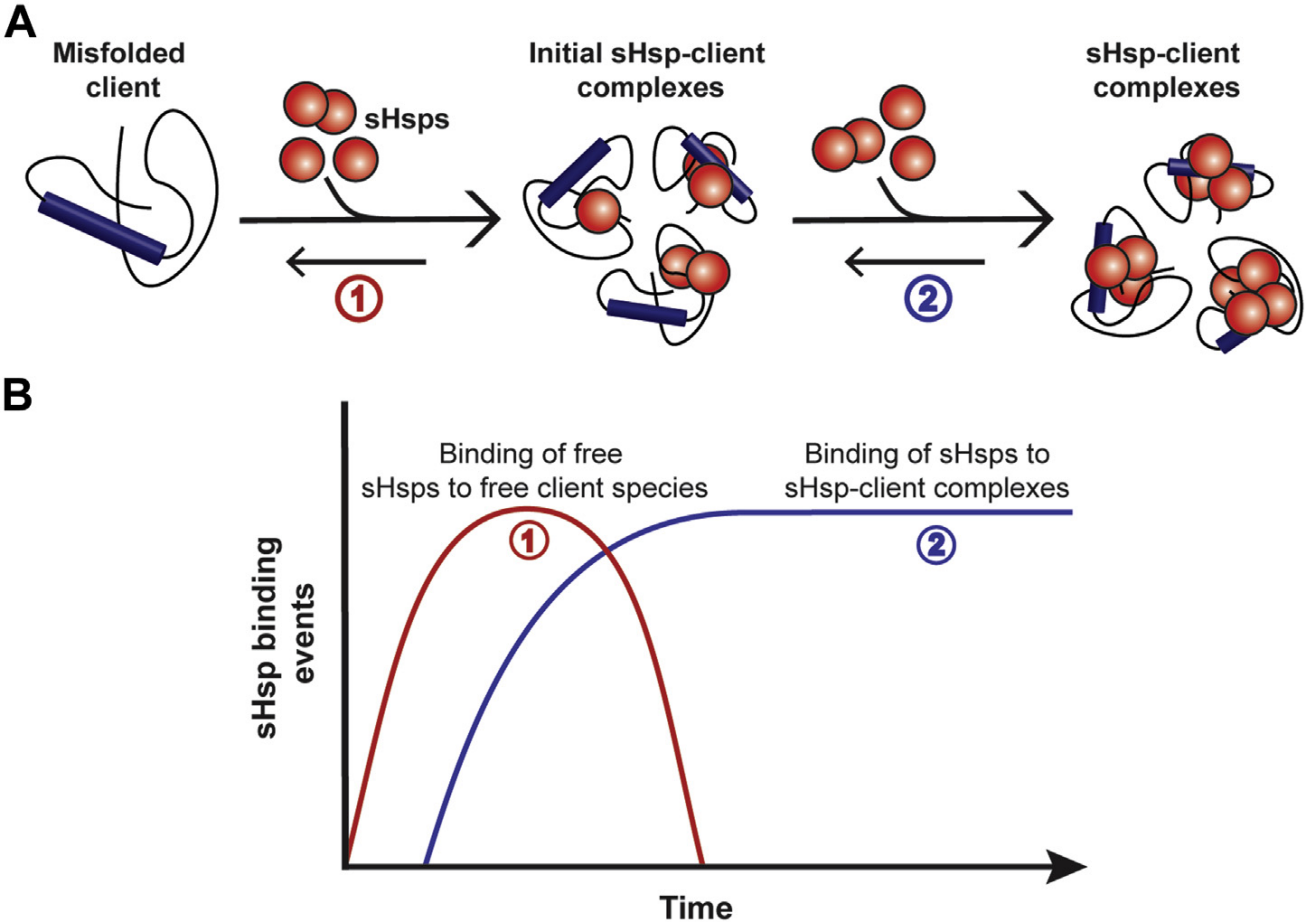
**Schematic of two-step mechanism of sHsp–client complex formation.***A*, smaller free sHsps initially recognize and stably bind free misfolded client proteins (1) allowing for subsequent binding of additional free sHsps subunits to form larger sHsp–client complexes (2). *B*, theoretical binding events of sHsp subunits over time showing that initial binding of free sHsps to free clients increases over time (1) until all the misfolded client is bound and additional free sHsp subunits associate with these complexes (2) in order to form larger sHsp–client complexes. sHsp, small heat shock protein.

Taken together, our findings provide direct experimental evidence for a two-step mechanism of sHsp–client complex formation that is in accordance with current models of sHsp chaperone action (Fig. 7) (23, 48, 49, 50). First, small sHsp species recognize and stably bind to misfolded client proteins and then these complexes grow through the subsequent addition of additional sHsp subunits onto the newly formed complexes until such a time that the system reaches equilibrium between bound and unbound sHsps and no further growth of the complexes occurs. Thus, the sHsp–client protein complexes we have characterized here are the building blocks of the high-molecular-mass complexes observed using other techniques (such as SEC) in which the sHsp is typically present at higher concentrations than we have used in the single-molecule fluorescence assay. Other than the concentration of the sHsp, the rate of association and dissociation of sHsp subunits from client complexes determines their maximum size. The actual size and the ratio of the sHsp–client protein complexes that are formed may vary for different client proteins. In the cellular context, factors that act to increase the rate of subunit exchange—*e.g.*, phosphorylation (13) or sHsp levels (*e.g.*, as occurs under conditions of cellular stress)—facilitate an increase in chaperone capacity through the provision of increased levels of “active” sHsps. At any given time, the optimum cellular level of sHsps occurs when the amount of the chaperone active species is sufficient to ensure that misfolded clients are stabilized in sHsp–client complexes. The potential for the formation of mixed sHsp hetero-oligomers places another level of complexity and control on sHsp chaperone action in cells.

A two-step mechanism of chaperone action is consistent with data obtained for plant sHsps (23) and the interaction of human αA-crystallin (HSPB4) with client proteins (51). Therefore, this is likely to be a universal functional mechanism of sHsps chaperone action. Future studies employing similar single-molecule fluorescence-based approaches to study the chaperone action of other polydisperse sHsps, such as Hsp27, will provide further insight into if this is indeed the case. Furthermore, similar studies that employ different client proteins would reveal whether the model of sHsp function described in this work is a general mechanism of sHsp–client interactions. Determining the precise molecular mechanisms of sHsps action is crucial to understanding how these molecular chaperones function to protect the cell from protein misfolding and their overall role in the cellular proteostasis network.

## Experimental procedures

### Materials, protein expression, and purification

All materials in this work were purchased from Sigma-Aldrich (St Louis, MO, USA) or Amresco (Solon, OH, USA) unless otherwise stated. The pET28a bacterial expression vector, containing human αBc wild-type (αBc_WT_) or mutant αBc_C176_, was used for expression of the recombinant proteins (Genscript, Piscataway, NJ). The mutant αBc_C176_ was engineered to contain an additional cysteine (compared with αBc_WT_) at the extreme C terminus to facilitate the site-specific covalent attachment of a fluorescent dye. Plasmids were transformed into competent *Escherichia coli* (*E. coli*) BL21 (DE3) cells. The αBc variants were purified as described previously (52) and stored at −20 °C.

CLIC1_C24_ in the pET24a vector was produced *via* site-directed mutagenesis of the wild-type genes (Genscript, Piscataway, NJ). The CLIC1_C24_ construct used in this study contained a mutation of one of the native tryptophan residues to phenylalanine (W23F) and mutations of five of the native cysteines to alanines (C59A, C89A, C178A, C191A, and C223A); the remaining cysteine (C24) was not modified so it could be exploited for site-specific fluorescent labeling. CLIC1_cysL_ in the pET24a vector was a kind gift from Dr Sophie Goodchild (Macquarie University, Australia). The CLIC1_cysL_ construct contained mutations of four of the native cysteins to alanines (C89A, C178A, C191A, and C223A); the remaining two cysteins (C24 and C59) were not modified. The pET24a vectors containing the CLIC1 variants (CLIC1_C24_ or CLIC1_cysL_) were each transformed into *E. coli* BL21 CodonPlus (DE3) RIPL cells, and recombinant protein expression was induced by the addition of 0.1 mM IPTG and overnight incubation at 18 °C. The cells were then harvested by centrifugation at 5000*g* for 10 min at 4 °C and the pellet stored at −20 °C. Cells were resuspended in 50 mM Tris-base (pH 8.0) containing 100 mM NaCl, 0.5 mg/ml lysozyme, and EDTA-free cocktail protease inhibitor, incubated for 20 min at 4 °C and then sonicated to further lyse cells and shear DNA. The cell lysate was then clarified by centrifugation twice at 24,000*g* for 20 min, passed through a 0.45-μM filter, and applied to a 5-ml HisTrap Sephadex column (GE Healthcare, USA) equilibrated in 50 mM Tris-base (pH 8.0) containing 5 mM imidazole and 300 mM NaCl. The bound recombinant protein was then eluted with 500 mM imidazole and loaded onto an s75 Superdex size-exclusion column equilibrated in 50 mM phosphate buffer (pH 7.4). The recombinant protein was concentrated, snap-frozen in liquid nitrogen, and stored at −20 °C until use. The SOD1 used in this work was a gift from Prof. Justin Yerbury (University of Wollongong, Australia).

### *In vitro* amorphous aggregation assays

*In vitro* aggregation assays were performed to assess the ability of αBc_WT_ and αBc_C176_ to inhibit the amorphous aggregation of CLIC1_cysL_ or CLIC1_C24_. CLIC1 (either 50 μM for CLIC1_cysL_ or 30 μM for the more destabilized CLIC1_C24_ isoform) was incubated in 50 mM phosphate buffer (pH 7.4) supplemented with 10 mM DTT in the presence or absence of varying molar ratios of αBc (between 1:0.5 and 1:64, αBc:CLIC1). CLIC1 incubated in the presence of SOD1 or ovalbumin at a 1:0.5 molar ratio (SOD1/Ova:CLIC1) acted as a control for the chaperone-specific inhibition of CLIC1 aggregation. Samples were prepared in duplicate in a Greiner Bio-One 384-well microplate (Greiner Bio-One, Freickenhausen, Germany) and sealed to prevent evaporation. The aggregation of CLIC1 was monitored by measuring the light scatter at 340 nm using a FLUOstar Optima plate reader at 37 °C for 20 h. To quantify the ability of the αBc variants to prevent CLIC1 aggregation, the percent inhibition of aggregation was calculated using the formula: % inhibition = ((Δ*Ic* − Δ*Is*)/Δ*Ic*) × 100, where Δ*Ic* and Δ*Is* are the change in absorbance in the absence and presence of chaperone at the end of the assay, respectively. The percent inhibition of aggregation afforded by the αBc variants is reported as the mean ± SD of three independent experiments.

### Analytical SEC and SDS-PAGE

Further characterization of the interaction between CLIC1_cysL_ and αBc_WT_ was achieved by analyzing samples by SEC at the end of the aggregation assays. Samples containing CLIC1_cysL_ (50 μM) in the presence or absence of αBc_WT_ (100 μM) were collected immediately following incubation and centrifuged at 20,000*g* for 10 min to remove any insoluble protein. Supernatants were then collected and loaded (80 μl) onto a Superdex 200 HR 10/300 GL column (GE Healthcare, UK) pre-equilibrated in 50 mM phosphate buffer (pH 7.4) and calibrated using Bio-Rad gel filtration standards (USA). Samples were eluted at 0.5 ml/min, and an in-line UV detector was used to monitor the elution of proteins from the column *via* their absorbance at 280 nm. Fractions corresponding to peaks on the chromatogram were collected and mixed with an equal volume of reducing sample buffer such that the final concentration of 2-mercaptethanol was 2.5% (v/v). These samples were subsequently heated at 95 °C before being run on a 12% (v/v) acrylamide gel for analysis *via* SDS-PAGE.

### Fluorescent labeling of proteins

For smFRET experiments, CLIC1_C24_ was labeled with an Alexa Fluor 555 donor maleimide fluorophore (AF555-CLIC1_C24_), and αBc_C176_ was labeled with an Alexa Fluor 647 maleimide acceptor fluorophore (AF647-αBc_C176_). For two-color single-molecule experiments, CLIC1_C24_ and αBc_C176_ were labeled with Alexa Fluor 647 and Alexa Fluor 488 maleimide fluorophores, respectively. Proteins were fluorescently labeled as previously described with some modifications (53). Briefly, proteins to be labeled were incubated in 5 mM tris(2-carboxyethyl)phosphine and 70% (w/v) ammonium sulfate powder and placed on a rotator at 4 °C for 1 h. Proteins were then centrifuged, and the pellet was resuspended in degassed buffer A (100 mM Na_2_PO_4_ (pH 7.4), 200 mM NaCl, 1 mM EDTA, 70% (w/v) ammonium sulfate). The protein was centrifuged, and the washed pellet was resuspended in buffer B (100 mM Na_2_PO_4_ (pH 7.4), 200 mM NaCl, 1 mM EDTA) containing a 5-fold molar excess of maleimide-conjugated fluorophore. The protein was then incubated on a rotator at room temperature for 3 h. Following the coupling reaction, excess dye was removed by gel filtration chromatography using a 7 K MWCO Zebra Spin Desalting column equilibrated in 50 mM phosphate buffer (pH 7.4). The concentration and degree of labeling was calculated for AF647-CLIC1_C24_ (96%), AF555-CLIC1_C24_ (82%), AF647-αBc_C176_ (77%), and AF488-αBc_C176_ (>95%) by UV absorbance or denaturing mass spectrometry (Table S1). The proteins were stored at −20 °C until use.

### Coverslip preparation and immobilization of samples for smFRET and two-color TIRF microscopy

Microfluidic flow cells were constructed by placing polydimethylsiloxane lids on 24 × 24-mm coverslips that had been PEG-biotin-functionalized (54). Coverslips were functionalized by treatment with 100% ethanol and 5 M KOH, before aminosilanization was carried out in a 1% (v/v) (3-aminopropyl) triethoxysilane (Alfa Aesar, UK) solution. PEGylation of coverslips was performed by incubating coverslips with 1:10 mixture of biotinPEG-SVA and mPEG-SVA (Laysan Bio, AL) prepared in 50 mM 3-(N-morpholino) propanesulfonic acid (pH 7.5) solution for 3 h. Coverslips were further functionalized by an additional PEGylation overnight before being stored under nitrogen gas at −20 °C. Inlets and outlets in the polydimethylsiloxane were prepared using PE-20 tubing (Instech, PA, USA) that allowed washing and addition of samples onto the coverslip surface. Neutravidin (125 μg/ml) was incubated in the flow cell for 10 min and washed with 50 mM phosphate buffer (pH 7.4) supplemented with 6-hydroxy-2,5,7,8-tetramethylchroman-2-carboxylic acid (6 mM, TROLOX) (imaging buffer). To help prevent nonspecific interactions of proteins with the coverslip surface, the microfluidic channel was blocked with 2% (v/v) Tween-20 for 20 min (55) and then washed extensively with imaging buffer. To facilitate immobilization of His-tagged CLIC1 to the coverslip surface, anti-6X His-tag antibody (1 μg/ml) was incubated in the flow cell for 10 min. Finally, preformed CLIC1–αBc complexes were diluted 1:1000, incubated in the flow cell for 10 min, and washed with imaging buffer to remove unbound proteins. To reduce blinking and unavoidable photobleaching of fluorescent dyes during imaging, an oxygen scavenger system (OSS) consisting of protocatechuic acid (2.5 mM) and protocatechuate-3,4-dioxygenase (50 nM) in imaging buffer was introduced into the flow cell prior to image acquisition.

### smFRET sample preparation, instrument setup, and data analysis

To confirm that αBc_C176_ formed complexes with aggregating CLIC1_C24_, smFRET experiments were performed. AF555-CLIC1_C24_ (1 μM) was incubated in the presence of AF647-αBc_C176_ (2 μM) for 20 h at 37 °C in 50 mM phosphate buffer (pH 7.4). The sample was then diluted 1:1000 in imaging buffer and immediately loaded into a flow cell for TIRF microscopy. Single-molecule measurements were performed at room temperature (approx. 20 °C) on a custom-built TIRF microscope with a sapphire green (532 nm) laser that has been previously described (56). Images were acquired every 200 msec, and single-molecule fluorescence intensity time trajectories from multiple fields of view (FOVs) were generated and analyzed using a Matlab-based software program (MASH-FRET) (57). Donor leakage into the acceptor channel was corrected during image analysis.

### Two-color TIRF microscopy instrument setup and data acquisition

Samples were imaged at room temperature (approx. 20 °C) using a custom-built total internal reflection fluorescence microscope system constructed around an inverted optical microscope (IX70, Olympus, Tokyo, Japan). Samples were illuminated simultaneously by a solid-state 488-nm laser (0.75 W/cm^2^; 150 mW Sapphire 488 nm, Coherent, Santa Clara, CA, USA) and 637-nm laser (6.5 W/cm^2^; 140 mW Vortran, Sacramento, CA, USA), which were aligned and directed off a dichroic mirror (Di01-R405/488/561/635, Semrock, Rochester, NY, USA) to the back aperture of a 1.49 NA TIRF objective lens (100 x UApoN model, Olympus) mounted on the optical microscope. Fluorescence emission was collected by the same objective, and the returning TIRF beam was filtered by a dichroic mirror (Di01-R405/488/561/635, Semrock). Then, incoming emission signals were separated using a dual view of 635-nm cutoff dichroic filter (Photometric DV2) that split incoming emission signals into two and directed them to a charge-coupled device chip, allowing simultaneous imaging of two colors on each half of the same chip, and passed through appropriate band-pass filters (BLP01–488R for AF488 and BLP01–633R for AF647) onto a EM-CCD camera (ImageEM, Hamamatsu, Japan). Control of the hardware was performed using the microscopy platform Micromanager (NIH, USA), and the camera was in frame transfer mode at 5 Hz. Multiple single-molecule movies of each sample were recorded at different FOVs, with images taken every 200 msec. All excitation intensities were kept constant for all samples imaged.

### Single-molecule characterization of surface binding of heated CLIC1_C24_

To investigate the ability of heated fluorescently labeled CLIC1_C24_ to bind surface immobilized anti-His antibodies, AF647-CLIC_C24_ (1 μM) was incubated at 37 °C for 2 h in 50 mM phosphate buffer (pH 7.4). The sample was subsequently diluted 1:1000 into imaging buffer and immediately loaded into flow cells that had been incubated in the presence or absence of the anti-6X His-tag antibody (1 μg/ml). Following a 10-min incubation, the flow cells were washed with imaging buffer containing an OSS to remove unbound proteins and immediately imaged with the red (637 nm) laser. The number of foci per FOV and the fluorescent intensity of each focus were calculated. The number of foci per FOV for each treatment group is reported as the mean ± standard deviation (n = 12). The fluorescent intensity of CLIC1_C24_ species in the treatment groups are presented as violin plots showing the kernel probability distribution, median, and interquartile range.

In order to examine the binding efficiencies of folded and thermally destabilized CLIC1_C24_, AF555-labeled CLIC1_C24_ (1 μM) was incubated at 37 °C (heated) for 2 h in 50 mM phosphate buffer (pH 7.4). AF647-labeled CLIC1_C24_ (1 μM) was incubated with heated or nonheated AF555-CLIC1_C24_ (1 μM) in 50 mM phosphate buffer (pH 7.4) on ice for 5 min. Samples were diluted 1:1000 in imaging buffer and immediately loaded into flow cells constructed with functionalized coverslips containing a surface-immobilized anti-6X His-tag antibody. Samples were incubated for 10 min before being washed with imaging buffer containing an OSS. Samples were imaged with a red (637 nm) laser until all visible foci were photobleached followed by a green (532 nm) laser to prevent the chances of any FRET occurring between the two fluorescently labeled CLIC1_C24_ species. The number of AF647-CLIC1_C24_ and AF555-CLIC1_C24_ foci in each image was counted and corrected to account for differences in the labeling efficiencies of AF647-CLIC1_C24_ (86%) and AF555-CLIC1_C24_ (73%). These values were then used to calculate the relative abundance of each fluorescently labeled CLIC1_C24_ per FOV.

### Single-molecule two-color sample preparation

Two-color TIRF microscopy was used to characterize the complexes formed between αBc and CLIC1. To determine how the stoichiometries of αBc–CLIC1 complexes changed over time, 1 μM Alexa Fluor 647-labeled CLIC1_C24_ (AF647-CLIC1_C24_) was incubated in 50 mM phosphate buffer (pH 7.4) at 37 °C for 10 h in the presence of 2 μM Alexa Fluor 488-labeled αBc_C176_ (AF488-αBc_C176_). Aliquots were taken from the reaction at 0, 0.25, 0.5, 0.75, 1, 4, 8, and 10 h for single-molecule imaging. To examine the effect of chaperone concentration on the stoichiometries of αBc–CLIC1 complexes, AF647-CLIC1_C24_ (1 μM) was incubated under the same conditions as described above except in the presence of varying molar ratios of AF488-αBc_C176_ (0.25:1, 0.5:1, 1:1, 2:1, and 4:1 [αBc:CLIC1]) for 8 h. All samples were diluted 1:1000 into imaging buffer and immediately loaded into flow cells for imaging.

### Two-color total internal reflection fluorescence microscopy data and statistical analysis

Images were corrected for electronic offset and inhomogeneity of the excitation beam laser before intensity time trajectories were generated for all fluorescent molecules using custom-written scripts in Fiji (58). The initial fluorescence intensity (*I*_*0*_) was calculated by averaging the first 20 intensity values for all fluorescent proteins identified. Fluorescent trajectories of molecules with distinct photobleaching events for AF647-CLIC1_C24_ and AF488-αBc_C176_ were manually identified and were fit by change-point analysis (59, 60) to determine the fluorescence intensity of each single-photobleaching event (*I*_*s*_). These *I*_*s*_ values were then collectively fit to a Gaussian distribution from which the mean intensity of a single photobleaching event (*I*_*s-mean*_) was calculated. The I_s-mean_ values were then used to calculate the number of *FPP* using the equation *FPP = I*_*0*_*/I*_*s-mean*_. At each treatment point (timepoint or concentration), *FPP* for AF647-CLIC1_C24_ or AF488-αBc_C176_ were combined to determine oligomer size distributions. Herein oligomer size refers to the number of subunits of a given protein in an oligomer (*e.g.*, for a single complex that contains 5 AF647-CLIC1_C24_ subunits, the CLIC1_C24_ oligomer size for that complex is 5). These oligomer sizes are presented as violin plots showing the kernel probability distribution, median, and interquartile range for each treatment. As fluorophores can self-quench when present at high local concentrations, complexes that contained more than 20 subunits of CLIC1_C24_ or αBc_C176_ were excluded from this detailed analysis of subunit architecture. Importantly, the maximum proportion of species present in solution that could not be characterized in detail was 12%; this was for the sample containing αBc_C176_ and CLIC1_C24_ incubated for 10 h at 37 °C at a molar ratio of 2:1 (αBc_C176_–CLIC1_C24_) (Fig. S10*A*).

All plots were generated, and statistical analysis was performed, using Prism8 (GraphPad, CA, USA). Data were analyzed *via* student’s *t* test or an ANOVA with subsequent Kruskal–Wallis tests followed by Dunn’s multiple comparisons (*p* values are given, whereby a *p* value of less than 0.05 was considered statistically significant). Stoichiometries of complexes were calculated by pairing of colocalized *FPP* for AF647-CLIC1_C24_ and AF488-αBc_C176_. Heatmaps were generated in MATLAB using home-written scripts.

## Data availability

All data and source code used in this work are available on request from the authors.

## Supporting information

This article contains supporting information (61).

## Conflict of interest

The authors declare that they have no conflicts of interest with the contents of this article.
