# Supplementary material for: Single-molecule fluorescence-based approach reveals novel mechanistic insights into human small heat shock protein chaperone function
Source: J Biol Chem. 2020 Dec 10;296:100161. doi: 10.1074/jbc.RA120.015419 (PMC7921601; doi:10.1074/jbc.RA120.015419)
Supplement: Supplementary Figures and Table [file mmc1.pdf]

Supporting Information

Single-molecule fluorescence-based approach reveals novel mechanistic insights into human small heat shock protein chaperone function

Caitlin L. Johnston[1], Nicholas R. Marzano[1], Bishnu P. Paudel[1], George Wright[2], Justin L. P. Benesch[2], Antoine M. van Oijen[1*] and Heath Ecroyd[1*]

[1]Molecular Horizons and School of Chemistry and Molecular Bioscience, University of Wollongong, Wollongong, NSW 2522, Australia; Illawarra Health & Medical Research Institute, Wollongong, NSW 2522, Australia.
[2]Department of Chemistry, Physical and Theoretical Chemistry, University of Oxford, UK.

[*]Correspondence to Heath Ecroyd (heathe@uow.edu.au) or Antoine van Oijen (vanoijen@uow.edu.au)

Material included:

- Supplementary Table 1: Summary of labelling methods and efficiencies
- Experimental Procedures for Fig. S2, S3, S5 and S8
- Supplementary Figures 1-10

Supplementary Table 1. Summary of labelling methods and efficiencies

**Protein**	**Label/dye**	**Method**	**Wavelength**	**ε (mg^{-1} ml cm^{-1})**	**Labelling efficiency**
$CLIC1_{C24}$	Alexa Fluor 647-C_2-maleimide	UV abs	650 nm	0.55	96%
	Alexa Fluor 555-C_5-maleimide	UV abs	556 nm	0.55	82%
αBc_{C176}	Alexa Fluor 647-C_2-maleimide	UV abs	650 nm	0.83	77%
	Alexa Fluor 488-C_5-maleimide	Mass spec			>95%

* Method = method used to determine labelling efficiency (UV absorbance at wavelength specified or denatured mass spectrometry).

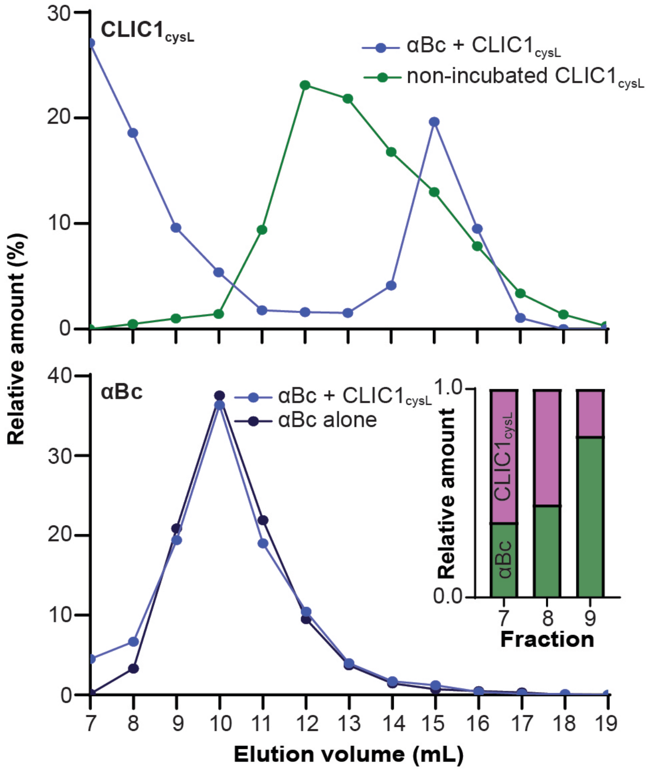

Supplementary Figure 1: Relative amount (%) of CLIC1$_{cysL}$ (*top*) and αBc$_{WT}$ in SEC fractions based on the intensity of protein staining in each lane of the SDS-PAGE gel presented in Fig. 1D. *Inset in the bottom panel* - The estimated ratios of CLIC1$_{cysL}$ to αBc in fractions 7-9 from the SEC column, based on relative staining intensities in the SDS-PAGE gel.

Experimental Procedures for Fig. S2

Cross-linking methodology
αBc$_{WT}$ (361 μM) in 50 mM phosphate buffer (pH 7.4) was incubated in the presence or absence of a 2.5, 5 or 10 molar excess of BS^3 cross-linker (Thermo Fisher Scientific, Waltham, MA) for 30 min at room temperature. Following incubation, non-reacted cross-linker was quenched with the addition of 50 mM glycine and removed using a 7K MWCO, 0.5 mL Zebra spin desalting columns (Thermo Fisher Scientific).

SDS-PAGE of cross-linked αBc
SDS-PAGE was conducted on a 12% (v/v) acrylamide gel using standard techniques. Samples containing cross-linked or control αBc were diluted 1 in 4 into a reducing sample buffer such that the final concentration of 2-mercaptethanol was 2.5% (v/v). Samples were heated at 95ºC before loading onto the gel.

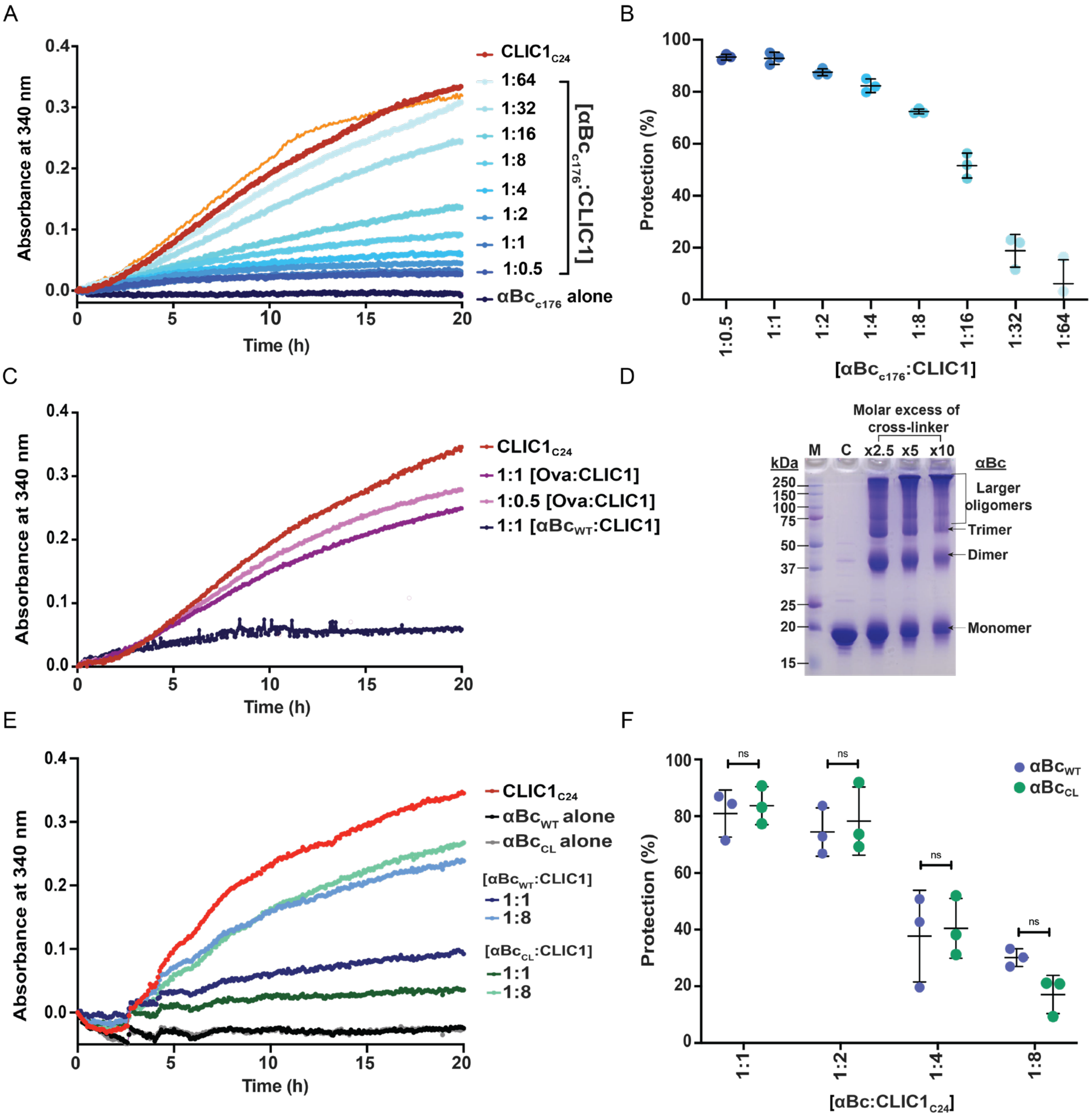

Supplementary Figure 2. αBc$_{c176}$ inhibits the amorphous aggregation of CLIC1$_{C24}$. Recombinant CLIC1$_{C24}$ (30 µM) was incubated in 50 mM phosphate buffer (pH 7.4) at 37°C in the presence or absence of varying molar ratios of αBc$_{C176}$:CLIC1$_{C24}$ ranging from 1:0.5 to 1:64 for 20 hours. The CLIC1$_{C24}$ aggregation was monitored via the change in light scatter at 340 nm over time. **(A)** A representative aggregation assay performed to assess the ability of αBc$_{C176}$ to inhibit the aggregation of CLIC1$_{C24}$. **(B)** The percentage protection afforded by varying molar ratios of αBc$_{C176}$ against CLIC1 aggregation, reported as mean ± standard deviation of three independent experiments (n = 3). **(C)** A representative aggregation assay demonstrating the chaperone-specific inhibition of CLIC1$_{C24}$ aggregation by αBc. The assay was performed by incubating recombinant CLIC1$_{C24}$ (30 µM) in 50 mM phosphate buffer (pH 7.4) at 37°C in the presence of αBc$_{WT}$ or a non-chaperone control protein ovalbumin (Ova) at varying molar ratios of αBc$_{WT}$/Ova:CLIC1$_{C24}$. **(D)** Analysis of αBc$_{WT}$ cross-linked (αBc$_{CL}$) with various molar excess of cross-linker (2.5, 5 or 10x) and non-cross-linked control (*C*) αBc$_{WT}$ via SDS-PAGE. **(E)** A representative aggregation assay performed by incubating recombinant CLIC1$_{C24}$ (30 µM) in 50 mM phosphate buffer (pH 7.4) at 37°C in the presence of αBc$_{WT}$ or αBc$_{CL}$ (x10 molar excess of cross-linker) at various molar ratios of αBc:CLIC1$_{C24}$ ranging from 1:1 to 1:8. **(F)** The percentage protection afforded by αBc$_{WT}$ or αBc$_{CL}$ at varying molar ratios of αBc:CLIC1$_{C24}$ against CLIC1$_{C24}$ aggregation, reported as mean ± standard deviation of three independent experiments (n = 3). Comparisons of αBc$_{WT}$ and αBc$_{CL}$ data at each molar ratio were performed via a student's t-test.

Experimental Procedures for Fig. S3

Mass photometry methodology

Preparation of the αBc samples for mass photometry mirrored that used in the single molecule assays. Thus, the samples of αBc were diluted from stock to 2 µM (monomer), in 50 mM filtered phosphate buffer (pH 7.4). These solutions were incubated for 30 min at room temperature, then 45 min on ice. Further dilution to 400 nM (monomer) was performed immediately before measurement. Coverslips were cleaned by sequential sonication in milli-Q water, isopropanol and milli-Q water (5 min each) before washing with ethanol and drying under a clean stream of nitrogen. Coverslips were then assembled into flow chambers as described previously (62).

Mass photometry measurements were made on a prototype of the ONE^{MP} (Refeyn Ltd). For the mass photometry measurement, 10-15 mL of sample was added to the flow chamber and recording initiated as soon as the sample stage returned to the focal position. Two 60-sec recordings were taken for each sample, at an effective frame rate of 318 Hz and an effective pixel size of 21.1 nm. The resultant movies were analysed using software written in-house according to previously described procedures (62) using stacks of 15 frames to produce the averages before division. To convert peak contrasts to mass, 15 standard proteins were measured on the same instrument under the same conditions, and the average contrast for each standard was plotted against the sequence mass to produce a linear calibration curve. The converted peak contrasts were then visualised in mass histograms (Supplementary Figure 2).

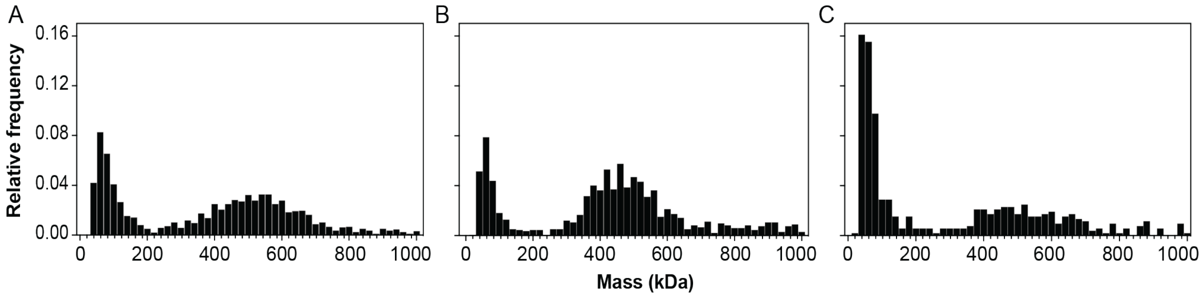

Supplementary Figure 3. αBc variants size distributions determined by mass photometry. Size distributions of **(A)** αBc_{WT}, **(B)** αBc_{C176} and **(C)** Alexa Fluor 488-labelled αBc_{C176}. Oligomers of the different forms of αBc are seen in the range 300-800 kDa, with some sub-oligomeric species observed for all three proteins at 400 nM (30 min after dilution from 2 μM at room temperature). Fluorescent labelling leads to some dissociation of the large oligomers, but the majority of AF-488 αBc_{C176} monomers remain in large oligomers.

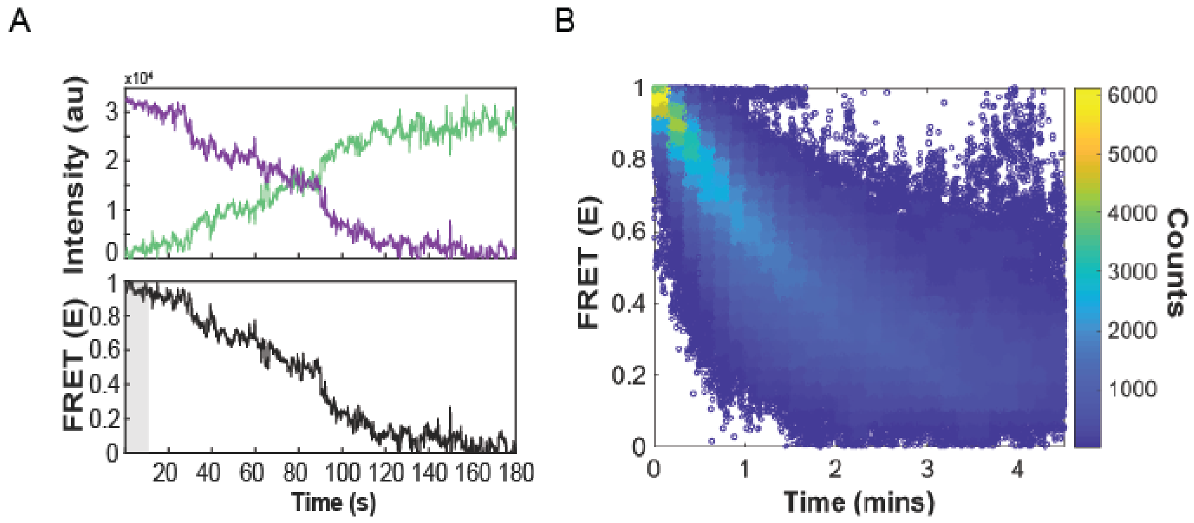

Supplementary Figure 4. αBc_{c176} and CLIC1$_{C24}$ form complexes that FRET. (A) A representative smFRET trace of the fluorescence intensity of the donor AF555-CLIC1$_{C59S}$ (*green*) and acceptor AF647- αBc_{C176} (*purple*) in complex over time. These intensity traces were used to calculate the FRET efficiency over time (*black*). *Grey area* represents the first 20 values that were used to construct FRET efficiency histogram for CLIC1$_{C24}$-αBc_{C176} complexes. **(B)** FRET efficiency heatmap (n = 421 molecules) of CLIC1$_{C24}$-αBc_{C176} complexes over time.

Experimental Procedure for Fig. S5

Negative stain transmission electron microscopy (TEM) methodology

AF488-αBc$_{C176}$ (2 μM) was incubated in the presence or absence of AF647-CLIC1$_{C24}$ for 20 hours at 37ºC in 50 mM phosphate buffer (pH 7.4). Following incubation, 4 μL of each sample was deposited onto 200 mesh copper grids with a 15-25 nm carbon substrate (Ted Pella, CA, USA) and incubated for 30 sec before being washed with water and stained with 2 % (w/v) uranyl acetate for 30 sec. Samples were viewed with a FEI Tecnai T-12 transmission electron microscope utilizing a Gatan Rio-4 CMOS based 4 Megapixel camera system (Gatan Inc., CA, USA).

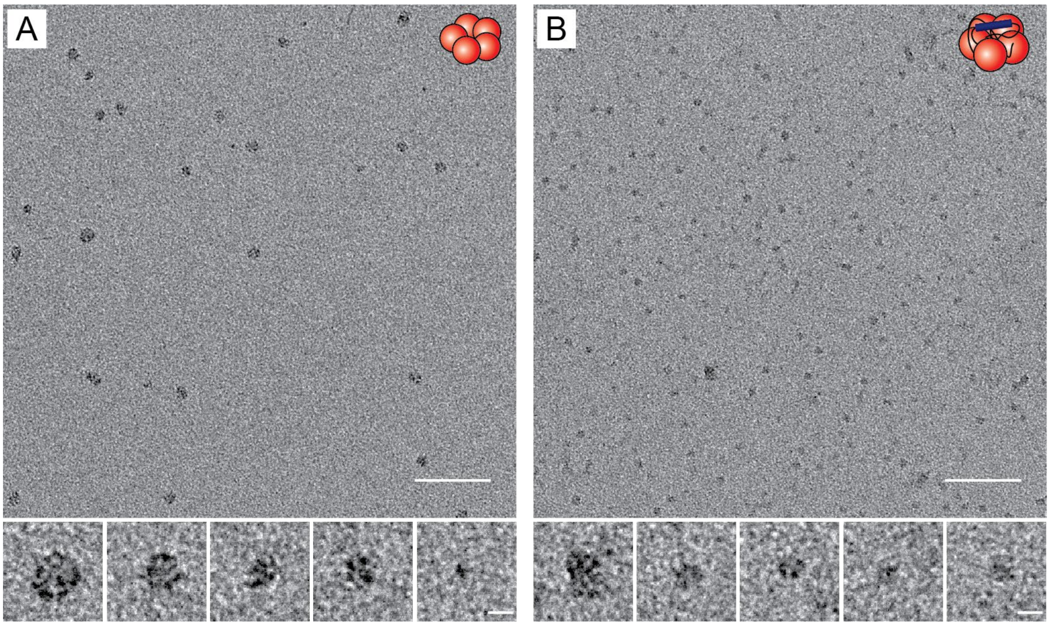

Supplementary Figure 5. Negative stain transmission electron microscopy (TEM) of AF488-αBc$_{C176}$ incubated in the presence or absence of AF647-CLIC1$_{C24}$. AF488-αBc$_{C176}$ (2 μM) was incubated in the presence or absence of AF647-CLIC1$_{C24}$ for 20 hours at 37ºC in 50 mM phosphate buffer (pH 7.4). Following incubation samples were deposited onto an EM gird and imaged. The fields of negatively stained A488-αBc$_{C176}$ in the **(A)** absence or **(B)** presence of AF647-CLIC1$_{C24}$ showing particles of different sizes in each sample - *scale bars = 100 nm*. Small images (*below*) show particles present within each sample – *scale bar = 10 nm*.

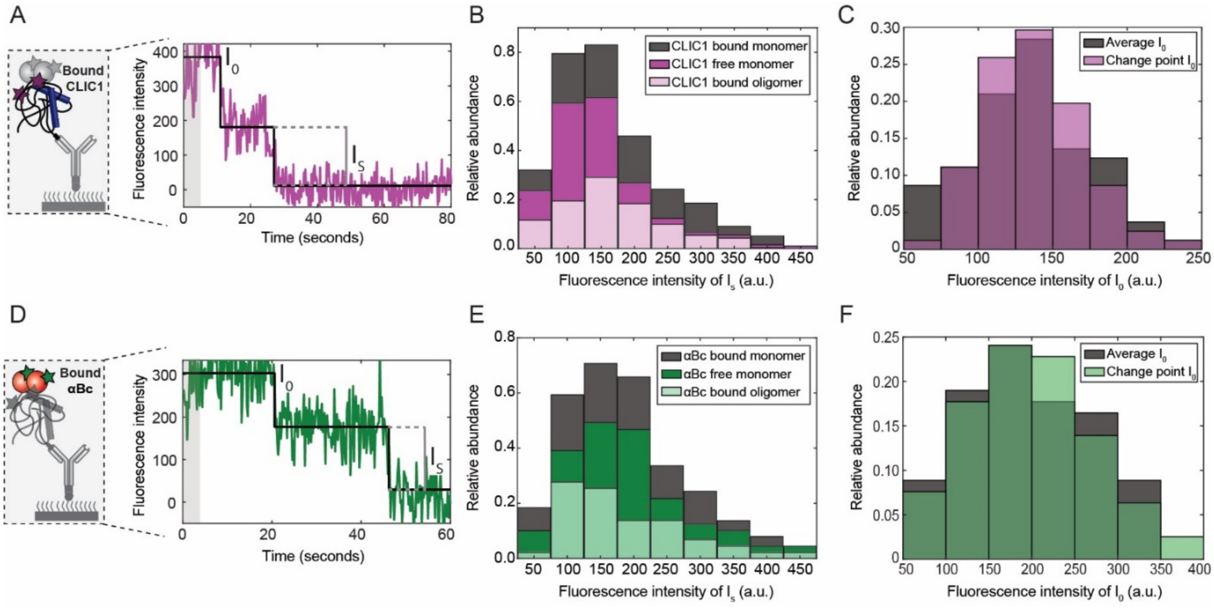

Supplementary Figure 6. The effect of complex formation on the fluorescence of single-photobleaching events (*I_s*) and calculation of initial fluorescence intensity (*I_0*) using CLIC1$_{C24}$ and αBc$_{C176}$ trajectories. Example time trace of the fluorescence intensity of **(A)** AF647-CLIC1$_{C24}$ or **(D)** AF488-αBc$_{C176}$ showing two distinct photobleaching steps fitted based on change-point analysis *(black)*, which was used to calculate I_0 (*change point I_0*) and I_s. The shaded area (*grey*) represents the first 20 fluorescence intensity values. Histogram comparing the relative abundance of I_s values calculated from manually selected trajectories of **(B)** AF647-CLIC1$_{C24}$ or **(E)** AF488-αBc$_{C176}$ when in complex (as a monomer or oligomer) or when freely bound to the surface as a monomer. Distribution of I_0 values of **(C)** AF647-CLIC1$_{C24}$ or **(F)** AF488-αBc$_{C176}$ calculated via averaging of the first 20 intensity values (*shown in grey on the time intensity traces*) or change point analysis.

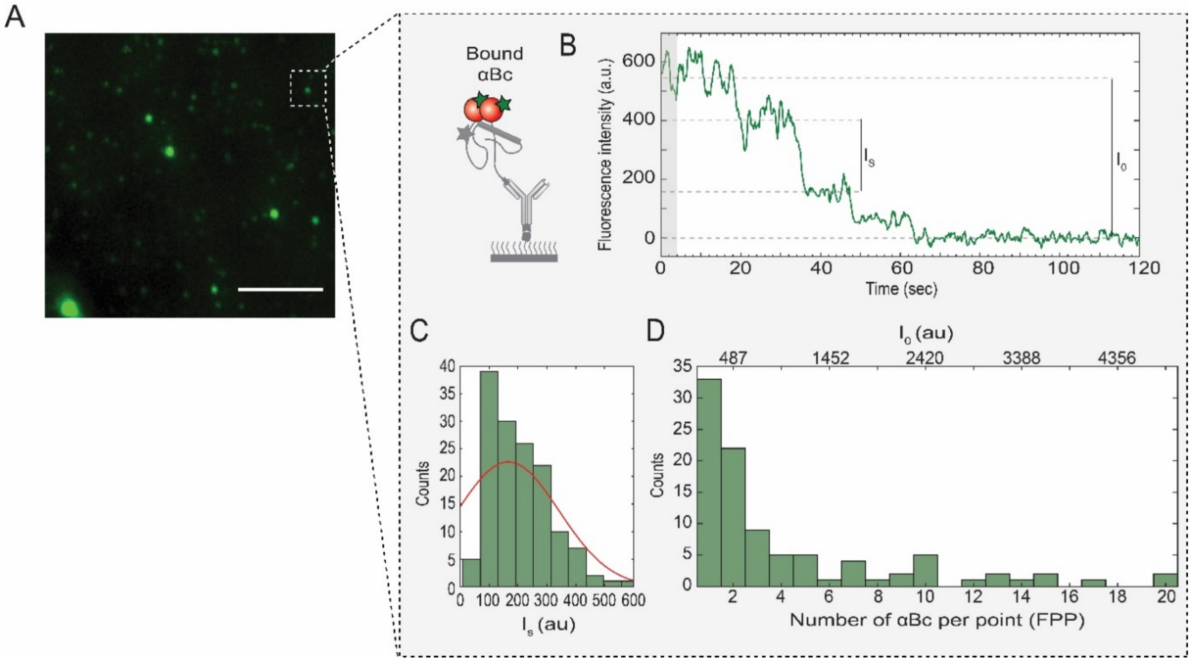

Supplementary Figure 7. Determination of the size distribution of αBc$_{c176}$ in complex with CLIC1$_{C24}$ using our single-molecule fluorescence-based approach. (A) Representative TIRF microscopy image of Alexa Fluor 488-labelled αBc$_{C176}$ in complex with CLIC1$_{C24}$ at 10 hr. *Scale bar* = 5 µm. The same TIRF microscopy image used in the lower panel of Figure 4A has been used here, in this case to highlight an AF488-αBc$_{C176}$ that is co-localised with CLIC1$_{C24}$. **(B)** Example time trace of the fluorescence intensity of AF488-αBc$_{c176}$ in complex with CLIC1$_{C24}$. *Grey areas* represent the first 20 fluorescence intensity values averaged to determine the initial intensity. **(C)** AF488-αBc$_{C176}$ intensity traces with distinct photobleaching steps were manually identified and fit to a change point analysis to calculate the fluorescent intensity of each single-photobleaching event (I_s). The I_s values were fit to a Gaussian distribution from which the mean intensity of a single photobleaching event ($I_{s\text{-}mean}$) was derived. **(D)** Example size distribution of showing the distribution of I_0 and number of αBc$_{C176}$ at 10 hr timepoint. The number of αBc$_{C176}$ was calculated by $I_0/I_{s\text{-}mean}$ for all the number of αBc$_{C176}$ in complex with αBc$_{C176}$ at 10 hr.

Experimental Procedures for Fig. S8

Cross-linking methodology

Alexa Fluor 488 αBc_{C176} (AF488-αBc_{C176}) was diluted from stock to either 2 µM or 10 µM in 50 mM phosphate buffer (pH 7.4). αBc_{C176}-CLIC1$_{C24}$ complexes were prepared by incubating 1 µM Alexa Fluor 647-labelled CLIC1$_{C24}$ (AF647-CLIC1$_{C24}$) in 50 mM phosphate buffer (pH 7.4) in the presence of 2 µM AF488-αBc_{C176} at 37°C for 2 hr. A non-heated control sample containing 1 µM AF647-CLIC1$_{C24}$ and 2 µM AF488-αBc_{C176} was also made up at room temperature. All samples were incubated at room temperature in the presence or absence of a 50 molar excess of BS^3 cross-linker (Thermo Fisher Scientific, Waltham, MA) for 30 min. Following incubation, non-reacted cross-linker was quenched with the addition of 50 mM glycine.

SDS-PAGE of cross-linked AF488-αBc_{C176}

SDS-PAGE was conducted on a 12% (v/v) acrylamide gel using standard techniques. Samples containing 10 µM AF488-αBc_{C176} that were incubated in either the presence or absence of cross-linker were mixed with an equal volume of reducing sample buffer such that the final concentration of 2-mercaptethanol was 2.5% (v/v). Samples were heated at 95ºC before loading onto the gel.

Single-molecule imaging of cross-linked samples

For AF488-αBc_{C176} samples, coverslips were cleaned by sequential sonication in 2 M KOH (15 min), 100% ethanol (15 min) and milli-Q water (5 min) before aminosilanisation was carried out in a 1% (v/v) (3-Aminopropyl) triethoxysilane (Alfa Aesar, UK) solution to increase the hydrophobicity of the surface for AF488-αBc_{C176} immobilisation. Cross-linked and control AF488-αBc_{C176} samples at either 2 µM or 10 µM were diluted to 10 nM in imaging buffer containing an oxygen scavenger system (OSS) consisting of protocatechuic acid (PCA, 2.5 mM) and protocatechuate-3,4-dioxygenase (PCD, 50 nM). Following dilution, samples were individually placed coverslips and single-molecule measurements were performed at room temperature on a custom built TIRF microscope with a solid-state 488 nm laser (setup described in the main text).

Samples containing both cross-linked and control αBc_{C176} and CLIC1$_{C24}$ were diluted 1:1000 into imaging buffer and incubated in flow cells made from PEG-biotin-functionalised coverslips and incubated for 10 min. Samples were subsequently washed with imaging buffer containing an OSS and were imaged using two-colour TIRF microscopy (described in the main text).

All sample images were acquired every 200 ms and single-molecule fluorescence intensity trajectories from multiple fields of view were generated and used to calculate the number of fluorescently labelled proteins per point (*FPP*) as previously described in the main text.

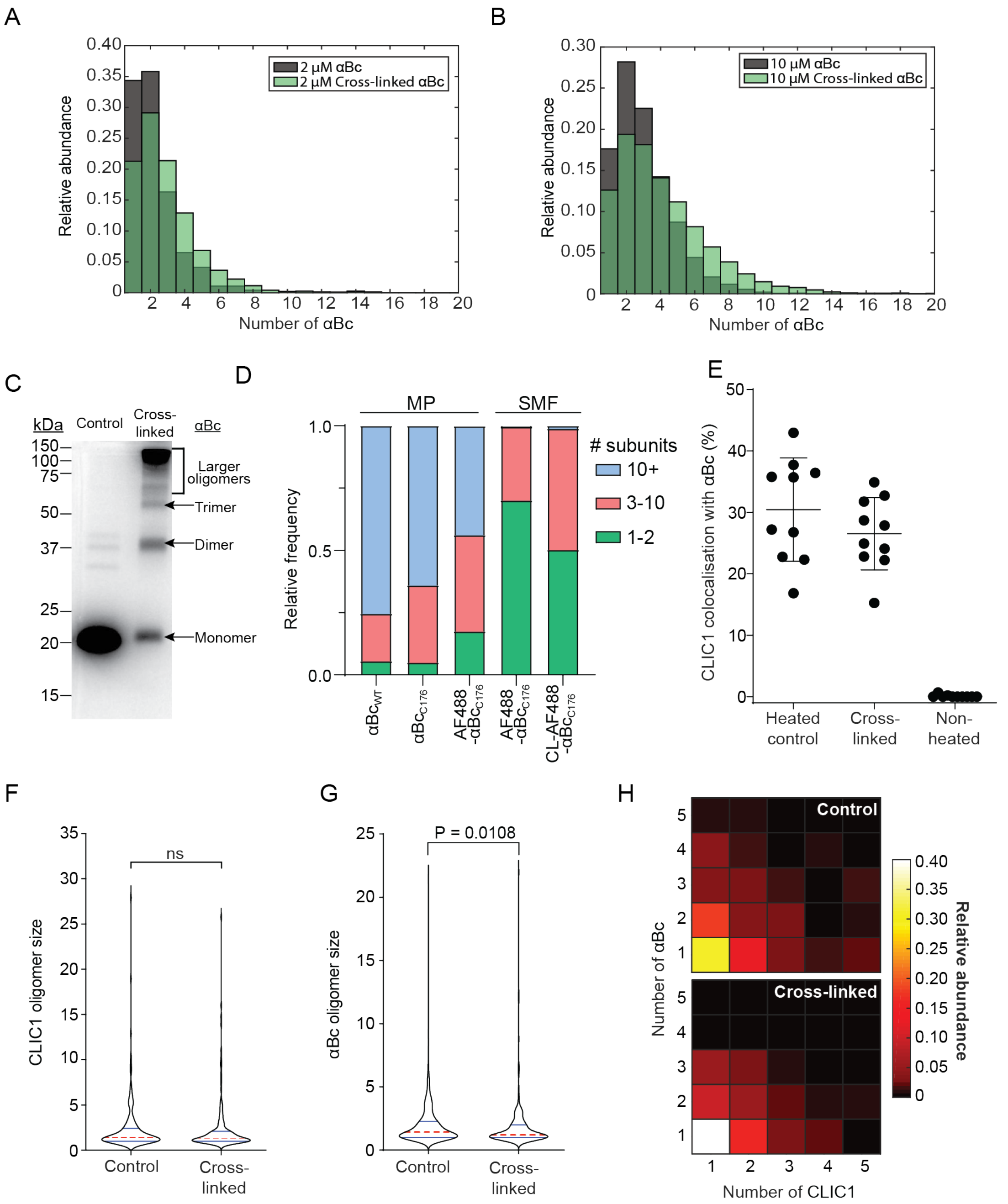

Supplementary Figure 8. The effect of concentration on the size of CLIC1$_{c24}$ and αBc$_{C176}$ examined at the single-molecule level using TIRF microscopy. Size distributions of cross-linked or control AF488-αBc$_{C176}$ at **(A)** 2 µM or **(B)** 10 µM AF488-αBc$_{C176}$. **(C)** 10 µM AF488-αBc$_{C176}$ control and cross-linked samples were analysed via SDS-PAGE. **(D)** The proportion of small (1-2), medium (3-10) or large (10+) oligomers detected using mass photometry (MP) (based on data presented in *Fig. S3*) or single-molecule fluorescence (SMF) of αBc$_{WT}$, αBc$_{C176}$, AF488-αBc$_{176}$ or cross-linked AF488-αBc$_{176}$ (CL-AF488- αBc$_{176}$) at 2 µM. **(E)** The percentage of AF647-CLIC1$_{C24}$ colocalised with AF488-αBc$_{C176}$ under various incubation and cross-linking conditions. All samples contained 1 µM AF647-CLIC1$_{C24}$ in the presence of 2 µM AF488-αBc$_{C176}$. The heated control was heated at 37°C for 2 hr but not cross-linked. The cross-linked sample was heated at 37°C and subjected to cross-linking. The non-heated sample was incubated at room temperature for 2 hr and cross-linked. Data is reported as mean ± SD of images collected. Violin plots showing the size distribution of colocalised **(F)** CLIC1$_{C24}$ and **(G)** αBc$_{C176}$ control and cross-linked samples. The violin plots show the kernel probability density (*black outline*), median (*red*) and interquartile range (*blue*). Comparisons of distributions was performed using Kruskal-Wallis test for multiple comparisons with Dunn's procedure (P values indicated). **(H)** Heat-maps showing the relative abundance of αBc$_{C176}$-CLIC1$_{C24}$ complexes and their stoichiometries in the cross-linked and control samples.

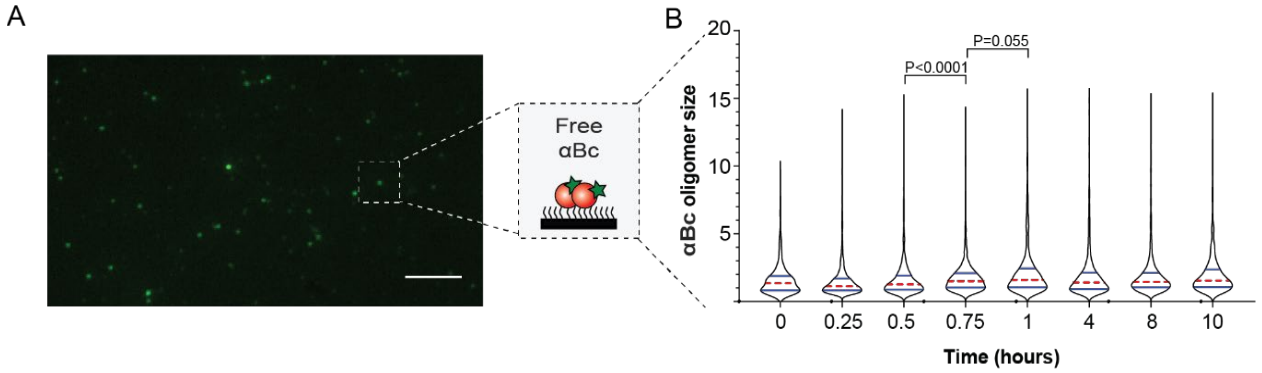

Supplementary Figure 9. Non-colocalised αBc$_{c176}$ increases in size over time. (A) Example image of non-specific binding of Alexa Fluor 488-labelled αBc$_{C176}$ to blocked coverslip surface. Alexa Fluor 488-labelled αBc$_{C176}$ (20 nM) was incubated in flow cell for 5 min, washed with imaging buffer and imaged using TIRF microscopy. *Scale bar* = 5 µm. **(B)** Size distributions of surface bound αBc$_{C176}$ at multiple time points over 10 hr. The violin plots show the kernel probability density (*black outline*), median (*red*) and interquartile range shown (*blue*). Result are representative of three independent experiments (n = 3).

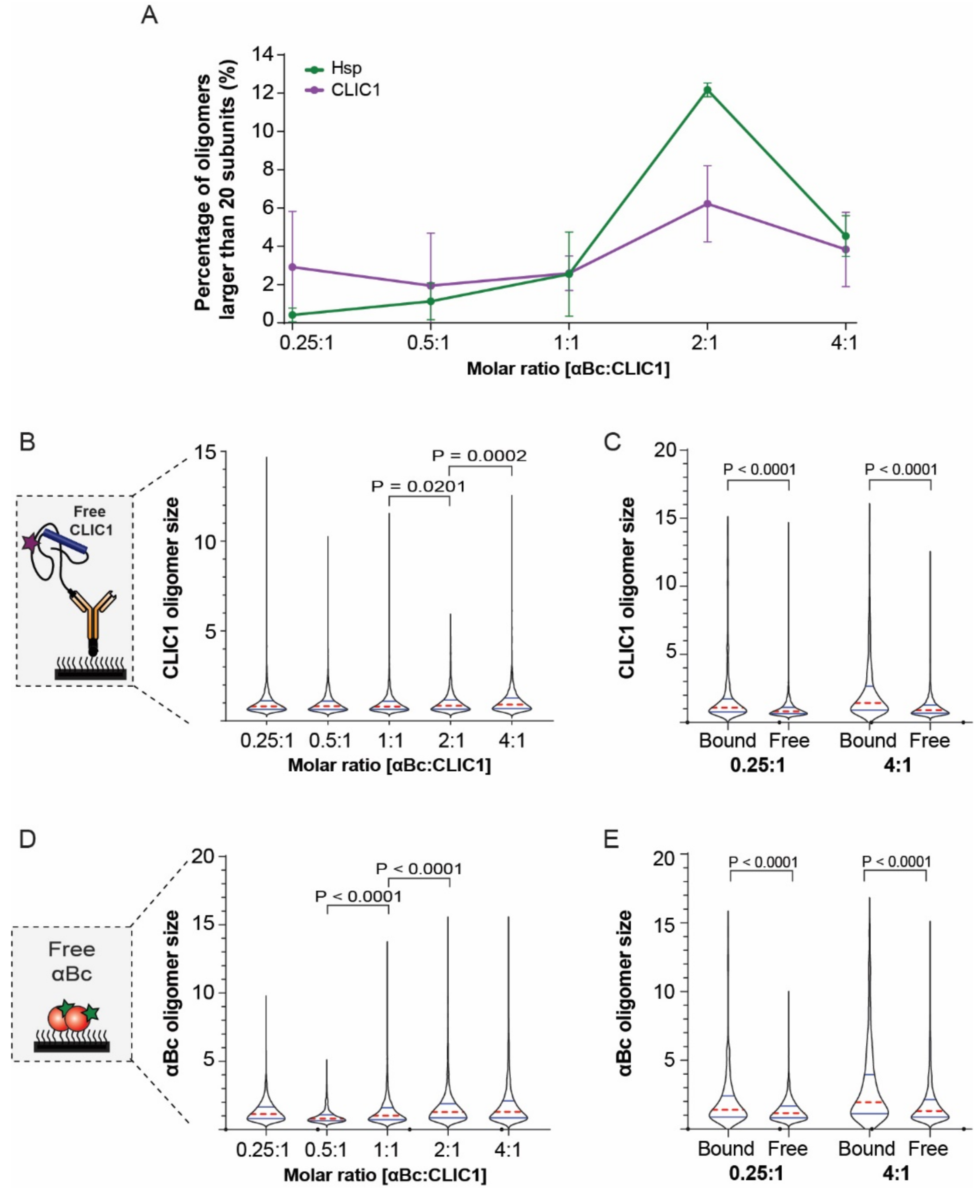

Supplementary Figure 10. CLIC1$_{C24}$ and αBc$_{c176}$ are significantly smaller when not in complex compared to when they are in complex. (A) Percentage of the population of Alexa Fluor 647-labelled CLIC1$_{C24}$ (*green*) or Alexa Flour 488-labelled αBc$_{C176}$ (*magenta*) oligomers in complex that contain more than 20 subunits with increasing molar ratios of αBc:CLIC1. Data is reported as mean ± SD of two independent experiments (n = 2). **(B)** Size distributions of free Alexa Fluor 647-labelled CLIC1$_{C24}$ not in complex with αBc$_{C176}$ at increasing molar ratios of αBc:CLIC1. **(C)** Size distributions of CLIC1$_{C24}$ bound to αBc$_{C176}$ compared to free CLIC1$_{C24}$ on the surface at 0.25:1 (*left*) and 4:1 (*right*) molar ratio. **(D)** Size distribution of non-specifically surface bound Alexa Fluor 488-labelled αBc$_{C176}$ at increasing molar ratios. **(E)** Comparison of size distributions of αBc$_{C176}$ bound to CLIC1$_{C24}$ and non-specifically bound (free) on the surface at 0.25:1 (*left*) and 4:1 (*right*) molar ratios [αBc$_{C176}$: CLIC1$_{C24}$]. The violin plots show the kernel probability density (*black outline*), median (*red*) and interquartile range shown (*blue*). Result are representative of two independent experiments (n = 2) and comparisons of distributions was performed using Kruskal-Wallis test for multiple comparisons with Dunn's procedure (P values indicated).
